# Tau filaments are tethered within brain extracellular vesicles in Alzheimer’s disease

**DOI:** 10.1038/s41593-024-01801-5

**Published:** 2024-11-21

**Authors:** Stephanie L. Fowler, Tiana S. Behr, Emir Turkes, Darragh P. O’Brien, Paula Maglio Cauhy, Isadora Rawlinson, Marisa Edmonds, Martha S. Foiani, Ari Schaler, Gerard Crowley, Sumi Bez, Elena Ficulle, Eliona Tsefou, Roman Fischer, Beth Geary, Pallavi Gaur, Chelsea Miller, Pasquale D’Acunzo, Efrat Levy, Karen E. Duff, Benjamin Ryskeldi-Falcon

**Affiliations:** 1 UK Dementia Research Institute at University College London, London, UK; 2 Medical Research Council Laboratory of Molecular Biology, Cambridge, UK; 3 Target Discovery Institute, Nuffield Department of Medicine, University of Oxford, Oxford, UK; 4 Taub Institute, Irving Medical Center, Columbia University, New York, NY USA; 5 Medical Research Council Protein Phosphorylation and Ubiquitylation Unit, University of Dundee, Dundee, UK; 6 Department of Neurology, Center for Translational and Computational Neuroimmunology, Columbia University Medical Center, New York, NY USA; 7 The Center for Genetic and Genomic Medicine, Hackensack University Medical Center, Hackensack, NJ USA; 8 Center for Dementia Research, Nathan S. Kline Institute for Psychiatric Research, Orangeburg, NY USA; 9 Department of Psychiatry, New York University Grossman School of Medicine, New York, NY USA; 10 Department of Biochemistry and Molecular Pharmacology, New York University Grossman School of Medicine, New York, NY USA; 11 Neuroscience Institute, New York University Grossman School of Medicine, New York, NY USA; 12 Present Address: Oxford-GSK Institute of Molecular and Computational Medicine, University of Oxford, Oxford, UK

**Keywords:** Alzheimer's disease, Neurodegeneration, Cryoelectron tomography, Protein aggregation, Cryoelectron microscopy

## Abstract

The abnormal assembly of tau protein in neurons is a pathological hallmark of multiple neurodegenerative diseases, including Alzheimer’s disease (AD). Assembled tau associates with extracellular vesicles (EVs) in the central nervous system of individuals with AD, which is linked to its clearance and prion-like propagation. However, the identities of the assembled tau species and EVs, as well as how they associate, are not known. Here, we combined quantitative mass spectrometry, cryo-electron tomography and single-particle cryo-electron microscopy to study brain EVs from individuals with AD. We found tau filaments composed mainly of truncated tau that were enclosed within EVs enriched in endo-lysosomal proteins. We observed multiple filament interactions, including with molecules that tethered filaments to the EV limiting membrane, suggesting selective packaging. Our findings will guide studies into the molecular mechanisms of EV-mediated secretion of assembled tau and inform the targeting of EV-associated tau as potential therapeutic and biomarker strategies for AD.

## Abstract (web summary)

Using cryo-electron tomography (cryo-ET) and proteomics, this study identifies the tethering of pathological tau filaments within defined brain extracellular vesicles in Alzheimer’s disease, shining light on the link between these vesicles and tau pathology.

## Main

Alzheimer’s disease (AD) is characterized by intraneuronal inclusions of hyperphosphorylated assembled tau protein, called neurofibrillary tangles, and extracellular plaques of assembled amyloid-β peptides. Tau assembles into two types of amyloid filament in neurofibrillary tangles, paired helical filaments (PHFs) and straight filaments (SFs), which are formed of two identical protofilaments with a C-shaped fold^1,2^. Tau assembly correlates with neuronal dysfunction and clinical symptoms in AD^3,4^, as well as a large group of neurodegenerative diseases collectively known as tauopathies^5,6^. Although wild-type tau assembles in most cases of tauopathy, rare mutations in the tau gene *MAPT* that lead to tau assembly and frontotemporal dementia demonstrate a causal role of tau assembly in neurodegeneration^7–9^.

In AD, tau assembly begins in the locus coeruleus and transentorhinal cortex and progresses over years to synaptically connected regions in the limbic system and neocortex^10,11^. Studies of tau assembly in cell and mouse models suggest that prion-like propagation may underlie this progression, including the intracellular trafficking, intercellular transfer and seeded aggregation of assembled tau^12–15^.

Extracellular vesicles (EVs) are lipid membrane-enclosed compartments that are released and taken up by many cell types, including neurons and glia^16^. They have been shown to selectively shuttle proteins, RNA, lipids and metabolites between cells^17^ and have been implicated in synaptic transmission in neurons^18,19^. EVs can be broadly categorized as exosomes or microvesicles (also known as ectosomes) based on their biogenesis pathway^17^. Exosomes are generated by budding of the endosomal membrane into the lumen of secretory multivesicular endosomes and are released into the extracellular space following fusion of multivesicular endosomes with the plasma membrane. Microvesicles bud directly from the plasma membrane. EVs can enter into recipient cells by endocytosis and deliver their contents to the cytoplasm by fusion with the limiting membranes of endo-lysosomes^17,20,21^.

Hyperphosphorylated assembled tau is associated with EVs isolated from the brain tissue and cerebrospinal fluid of individuals with AD^22–25^. Similar observations have been made for EVs from transgenic mice expressing mutant tau^24^. EVs from the central nervous system of individuals with AD seed tau assembly when added to cultured cells and when injected into the brains of tau transgenic mice^22,24–26^. EVs deliver assembled tau to the cytoplasm of recipient cells via the endo-lysosomal system^27^. Studies comparing non-EV-associated and EV-associated assembled tau have shown that EV-associated tau is more potent at seeding assembly^22,25,28,29^. These studies suggest that EVs have the potential to mediate the prion-like propagation of assembled tau in the human brain.

The molecular species and structures of assembled tau, including oligomers and filaments, and the identities of the EVs that they associate with are not known. In addition, how assembled tau associates with EVs, including if it is luminal or surface bound, is unclear. This information would provide insights into the molecular mechanisms governing the secretion of assembled tau within EVs. It will also inform AD biomarker discovery and therapeutic strategies targeting assembled tau.

Here, we isolated EVs from brain tissue of individuals with AD using density gradient centrifugation and profiled their protein composition using quantitative mass spectrometry. We confirmed the ability of the EVs to seed tau assembly in cultured cells and in vivo. We characterized the molecular species and structures of assembled tau associated with EVs using quantitative mass spectrometry, biochemical fractionation and cryo-electron microscopy (cryo-EM). Finally, we used cryo-electron tomography (cryo-ET) to investigate the association of assembled tau with EVs. Our results show that PHFs and SFs formed of truncated tau are tethered inside EVs enriched in endo-lysosomal proteins in the brains of individuals with AD.

## Results

### Isolation of EV populations from the brains of individuals with AD

**Fig. 1:**
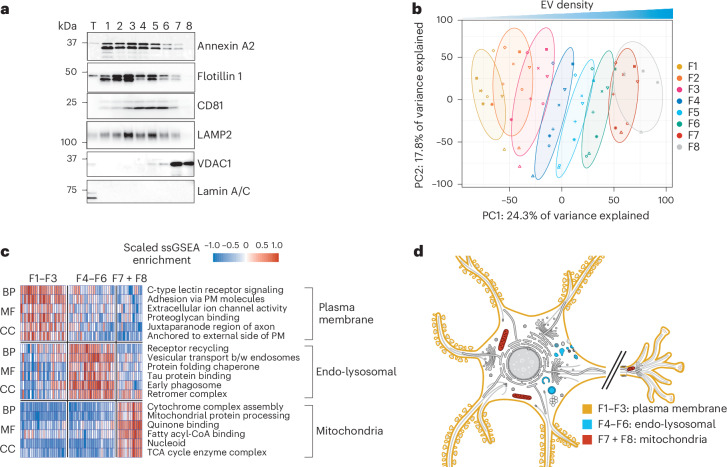
Proteomic characterization of EVs from brain tissue of individuals with AD. **a**, Representative immunoblot of total homogenate (T) and EV fractions 1–8 isolated from the brain tissue of individuals with AD using antibodies to annexin A2, flotillin 1, CD81, LAMP2, VDAC1 and lamin A/C; *n* = 5. **b**, Principal component (PC) analysis of quantitative mass spectrometry of EV fractions 1–8 from human AD brain samples. Symbols denote different cases; *n* = 8 for fractions 1–7 (F1–7) and *n* = 5 for fraction 8. Ellipses indicate 68% confidence intervals. **c**, Gene Ontology pathway enrichment analysis of quantitative mass spectrometry of grouped EV fractions 1–3, 4–6 and 7 and 8. Each column represents an individual case; *n* = 8. Per row scaled single-sample gene set enrichment analysis (ssGSEA) enrichment scores are shown; BP, biological process; MF, molecular function; CC, cellular component; PM, plasma membrane; b/w, between; TCA, tricarboxylic acid. **d**, Enriched biosynthetic subcellular localization of enriched proteins in each EV group using the SwissBioPics animal neuronal cell. Source data

**Extended Data Fig. 1:**
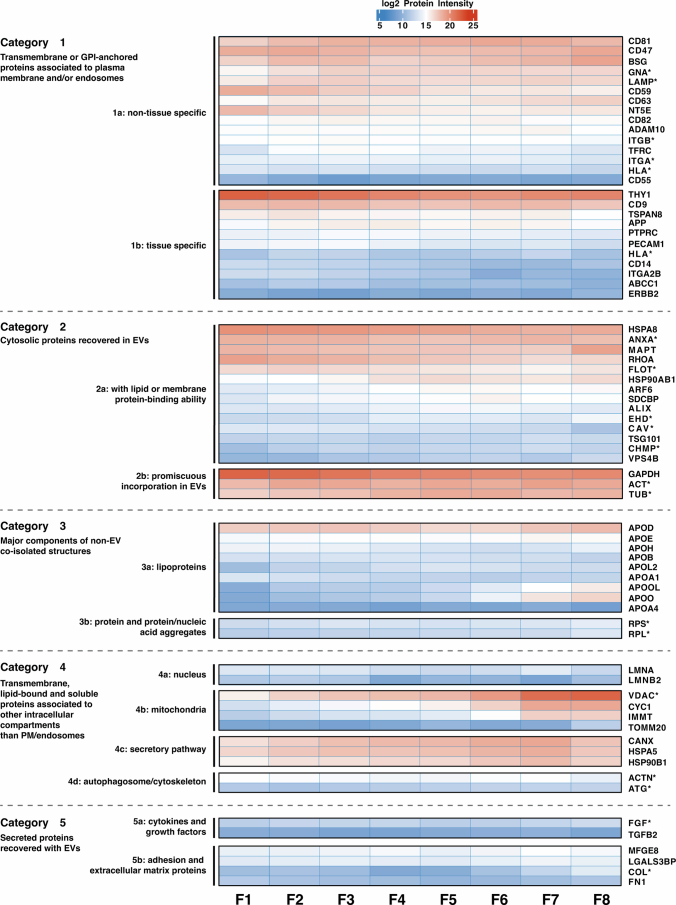
Quantitative mass spectrometry proteomics of EVs isolated from AD patient brain. Proteins are listed on the right-hand side and their Log2 protein intensity is shown for each fraction (F) 1–8. Proteins are grouped into categories according to Ref. ^32^. n = 8 patients.

**Extended Data Fig. 2:**
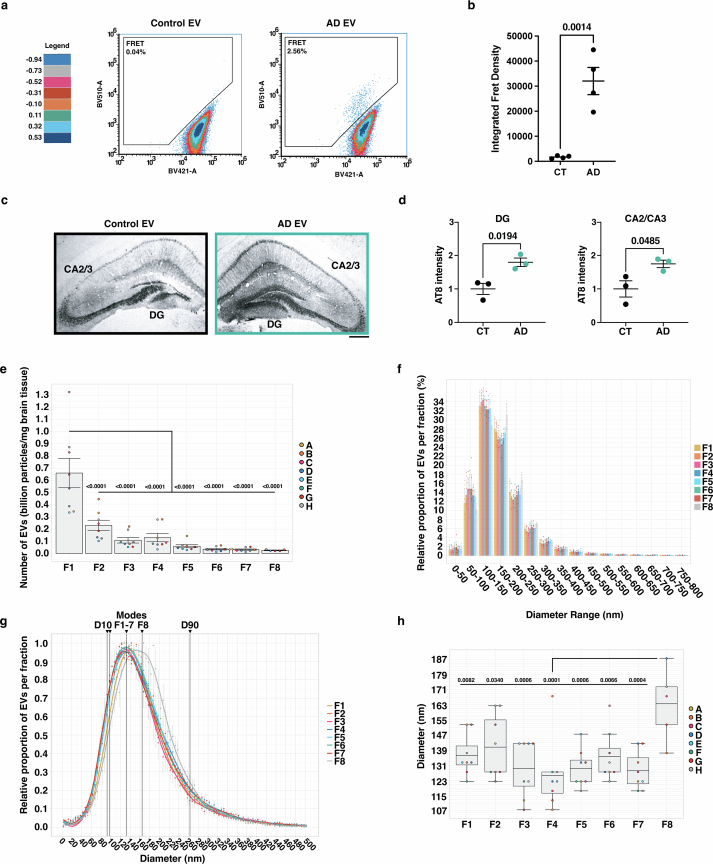
Tau seed competency and NTA characterisation of EVs isolated from AD brain. **a**, Representative FRET gating strategy for the Tau RD P301S FRET Biosensor cell line treated with 3 μg pooled neurologically-normal control and AD EVs. Positive tau seeding is indicated by the presence of BV510 fluorescence signal in the FRET gate. Colour scale indicates the log density of measured event abundance. **b**, Integrated FRET density (% cells in the FRET gate multiplied by the mean fluorescence intensity of the BV510 signal) of cells incubated for 48 h with 3 μg pooled neurologically-normal control and AD EVs, n = 4. Data are means of triplicate technical replicates, two-tailed unpaired t-test. **c**, Representative immunohistochemistry images of PS19 mouse hippocampal fields using anti-tau antibody AT8 (phospho-ser202 and -thr205 tau) two-months post stereotaxic injection of pooled EV fractions isolated from neurologically-normal control brain and AD brain into the DG, n = 3. Scale bar, 100 μm. **d**, Quantification of the mean AT8 intensity in the DG and the CA2/CA3 hippocampal fields of PS19 mice injected with EVs from CT and AD brain, n = 3, two-tailed unpaired t-test. **e**, Nanoparticle tracking analysis (NTA) of total number of EVs per fraction 1–8. Data are expressed as billions of particles per mg brain tissue, one-way ANOVA with Dunnett’s post-hoc to F1. **f**, NTA analysis of the relative proportion (%) of EVs assigned to the depicted diameter bins for each fraction 1–8. **g**, NTA analysis of EV diameter for each fraction 1–8 normalised to the mode of each distribution. D10 (F1-F7: 92.5 nm, F8: 97.5 nm) and D90 (262.5 nm for all fractions) values are means of each value across all fractions. **h**, NTA analysis of EV diameters expressed as the mode of each fraction 1–8 for each donor. Box plot centre lines indicate the mean value, lower and upper hinges represent the first and third quartiles, and whiskers extend from the hinges to a maximum of 1.5X the interquartile range, one-way ANOVA with Dunnett’s post-hoc to F8. **e-h**, n = 8 human donors for F1-F7, n = 5 for F8, means of triplicate technical replicates were plotted for each donor. **b,d,e-g**, Data are presented as mean values +/− SEM. **b,d,e,h**, *p*-values are shown above data points.

We isolated EVs from flash-frozen postmortem frontal and temporal cortex and hippocampal tissue from individuals with AD across eight fractions using density gradient centrifugation (Fig. 1 and Extended Data Table 1). Immunoblotting of the EV fractions compared to total brain homogenates demonstrated the enrichment of known EV-associated proteins^30^, including the microvesicle-enriched proteins annexin A2 and flotillin 1, the exosome-enriched tetraspanin CD81 and the lysosomal-associated membrane protein 2 (LAMP2; Fig. 1a). Intracellular proteins, such as lamin A/C were depleted. Quantitative mass spectrometry proteomics of the EV fractions mapped overwhelmingly to the Gene Ontology term ‘extracellular membrane-bounded organelle’ (GO:0065010, *P* = 4.941 × 10^−324^, g:Profiler). Using the five-component framework from the Minimal Information for Studies of Extracellular Vesicles (MISEV) for reporting claims about the protein content of EVs^31^, we observed enrichment of EV-associated proteins (categories 1 and 2) and depletion of common protein contaminants (categories 3 and 5), including abundant intracellular proteins such as lamin A/C, actin and Tomm20 (Extended Data Fig. 1 and Supplementary Data 1). We confirmed the ability of the EVs to seed tau assembly in the Tau RD P301S FRET Biosensor cell line (Extended Data Fig. 2a,b)^32^ as well in transgenic mice expressing human tau-P301S (PS19 line)^33^ 2 months following injection into the hippocampal dentate gyrus (Extended Data Fig. 2c,d).

Nanoparticle tracking analysis (NTA) showed that the majority of EVs were collected in the lightest density fraction 1 (Extended Data Fig. 2e). EVs in fractions 1–7 had similar size distributions, with average mode diameters of 132.1 nm ± 13.98 nm. By contrast, EVs in fraction 8 were significantly larger, with diameters of 163.5 nm ± 19.17 nm (Extended Data Fig. 2f,h). Across all fractions, 10% of EVs had diameters smaller than 92.5 nm (D10), and 10% had diameters larger than 262.5 nm (D90; Extended Data Fig. 2g), in agreement with previous studies of EV size distributions using NTA and cryo-EM^34,35^.

**Extended Data Fig. 3:**
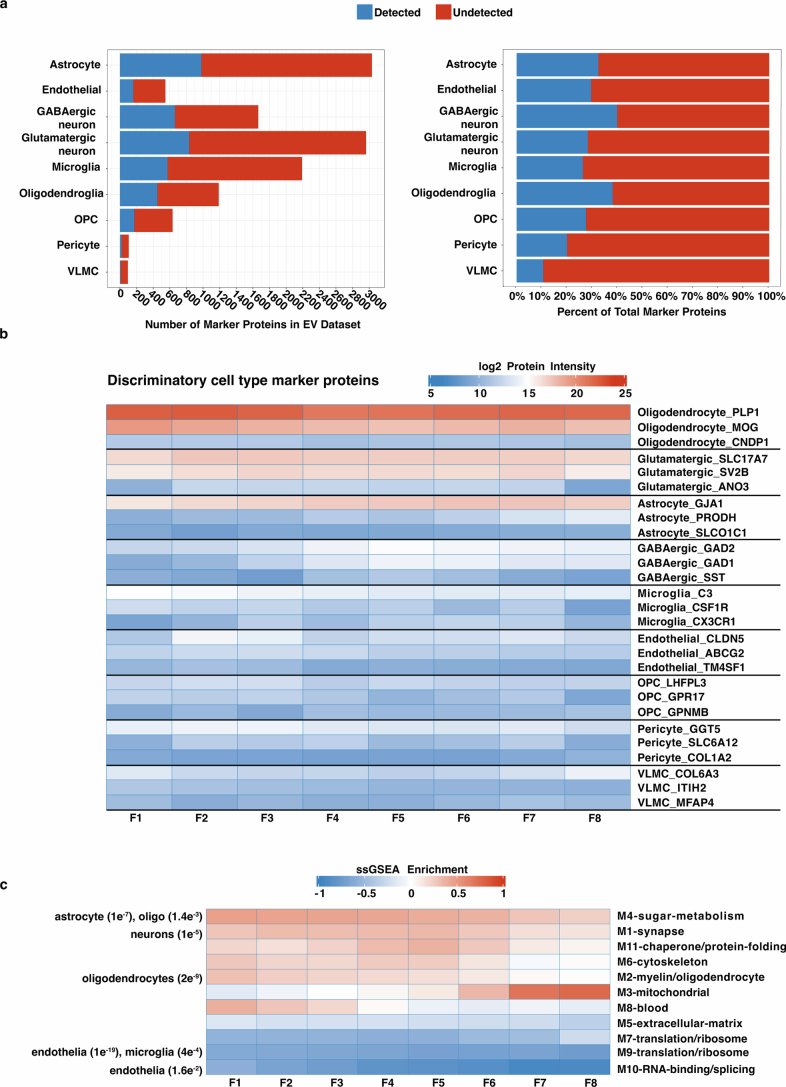
Cell type marker proteins identified in human AD brain EVs. **a**, Number (left) and percent (right) of cell type marker proteins identified in the full EV dataset. The most number of cell type specific proteins were identified for astrocytes, neurons, microglia and oligodendrocytes, however, we detected between 30–40% of total known marker proteins for all cell types except pericytes and VLMCs (vascular and leptomeningeal cells). **b**, The top three discriminatory marker proteins for each cell type displayed across fractions F1-F8. Cell types are ordered according to the mean log2 protein intensity detected across all fractions for the 3 marker proteins. **c**, Mapping of AD brain co-expression modules onto AD brain EV fractions. Modules are ordered in the heatmap according to the mean ssGSEA enrichment score across all density fractions. Where overlap of module proteins with known cell type markers was significant, the cell type and significance of overlap is reported on the left of the heatmap.

We developed an imputation strategy for the detection of enriched proteins among fractions in our quantitative mass spectrometry analysis (Fig. 1b–d and Supplementary Data 1). Fractions 1–3 were enriched in plasma membrane-associated proteins, suggesting a predominance of microvesicles. By contrast, fractions 4–6 were enriched in endo-lysosomal proteins, suggesting that they mainly contained exosomes. Fractions 7 and 8 were enriched in mitochondrial proteins, supported by immunoblotting showing that the mitochondrial voltage-dependent anion channel 1 was restricted to these fractions (Fig. 1a,c). EVs in fractions 7 and 8 are consistent with recently described mitovesicles^36^. We detected 10–40% of known marker proteins for human brain cell types (Extended Data Fig. 3a). Across all fractions, marker proteins for oligodendrocytes, glutamatergic neurons and astrocytes were the most highly enriched (Extended Data Fig. 3b). We also assessed recently reported AD-related coexpression modules^37^. EVs were enriched in sugar metabolism (M4), synapse (M1), chaperone/protein folding (M11), cytoskeleton (M6), myelin/oligodendrocyte (M2), mitochondrial (M3) and blood (M8) modules (Extended Data Fig. 3c). AD module proteins were differentially enriched across the EV fractions, consistent with the immunoblot and Gene Ontology analyses (Fig. 1a,c), and exhibited significant overlap with protein identities associated with astrocytes (M4), oligodendrocytes (M4 and M2) and neurons (M1). These results demonstrate that EV populations enriched in distinct organelle components can be isolated from postmortem AD brain tissue based on density.

### EVs from the brains of individuals with AD are associated with truncated tau

**Fig. 2:**
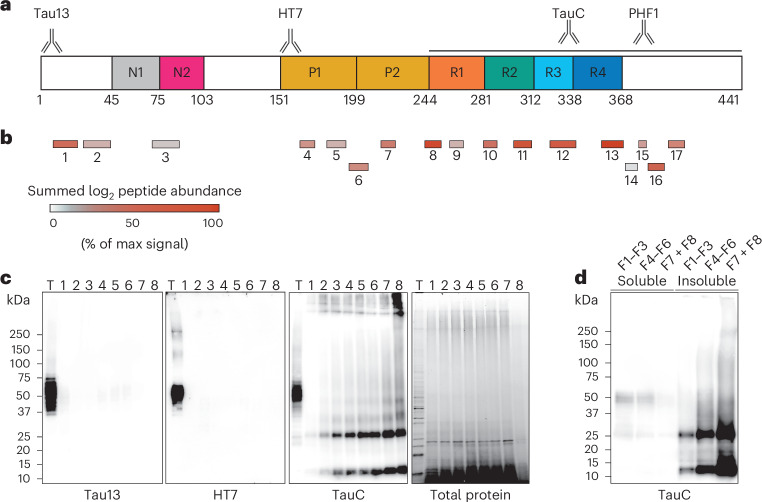
Mass spectrometry and immunoblot analyses of tau protein associated with EVs from the brains of individuals with AD. **a**, Schematic of the longest 2N4R tau isoform (441 amino acids) with epitopes shown for antibodies Tau13, HT7, TauC and PHF1 (anti-phospho-S396 and phospho-S404). The tau N-terminal inserts (N1 and N2), proline-rich regions (P1 and P2) and repeats (R1–R4) are shown. **b**, Tau peptides identified by quantitative mass spectrometry in EV fractions 1–8. Imputed, normalized and summed tau peptide label-free quantification intensities are shown. **c**, Representative immunoblot of total homogenate (T) and EV fractions 1–8 isolated from the brains of individuals with AD using antibodies Tau13, HT7 and TauC. Total protein loading control was visualized using a stain-free gel; *n* = 8. **d**, Representative immunoblot of total, sarkosyl-soluble and sarkosyl-insoluble fractions of pooled EV fractions 1–3, 4–6 and 7 and 8 from the brains of individuals with AD using antibody TauC; *n* = 3. Source data

We investigated the association between tau and the EV populations from the brain tissue of individuals with AD. Peptides encompassing the four tau repeats (residues 242–370) were enriched in our quantitative mass spectrometry analysis (Fig. 2a,b and Supplementary Data 1). Peptides from the extreme N terminus (residues 6–23), the proline-rich region and adjacent to the C-terminus of the repeat region were also detected (Fig. 2a,b and Supplementary Data 1). Immunoblotting of the EV fractions showed the presence of full-length and truncated forms of tau, the majority of which existed as approximately 12- and 24-kDa species (Fig. 2c). The truncated forms of tau were detected by an antibody raised to the C-terminal half of tau (residues 242–411; TauC) but not by antibodies to epitopes between the extreme N terminus (residues 2–18; Tau13) and the proline-rich region (residues 159–163; HT7; Fig. 2c).

**Extended Data Fig. 4:**
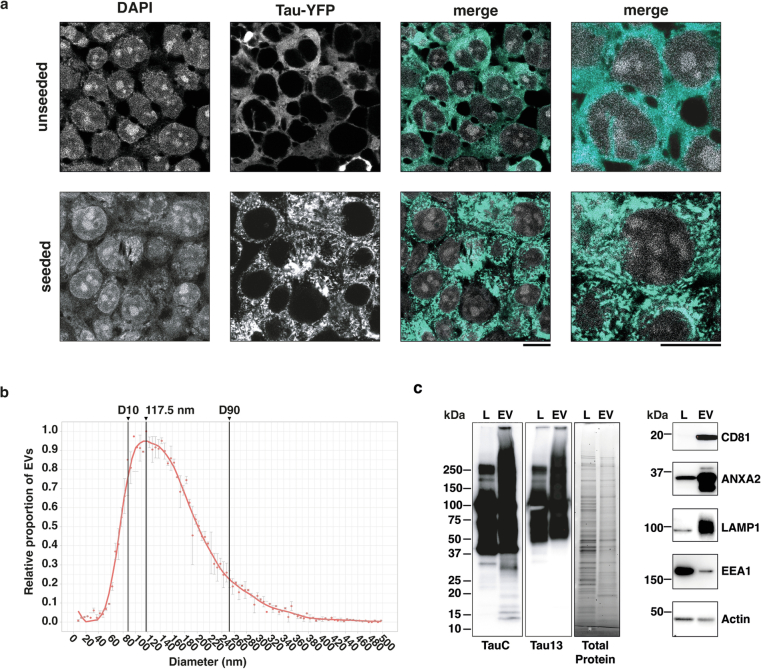
Characterisation of EVs isolated from the media of HEK cells expressing full-length assembled tau. **a**, Confocal imaging of HEK293T cells expressing P301S 1N4R tau fused to YFP (tau-YFP) immunolabelled using an antibody against YFP, representative n = 3. The seeded cell line was generated by the addition of tau PHFs isolated from AD human brain tissue. Scale bars = 10 μm. **b**, NTA of EVs isolated from seeded tau-YFP HEK293T cells. Mode diameter = 117.5 nm, D10 = 87.5 nm, D90 = 252.5 nm. Data are presented as mean values +/− SEM of 4 technical replicates, n = 1. **c**, Immunoblot of seeded tau-YFP HEK293T cell lysates and EVs isolated from culture media of seeded tau-YFP HEK293 cells using the anti-tau antibodies TauC and Tau13, and antibodies against CD81, annexin A2 (ANXA2), LAMP1, EEA1 and actin, n = 2. Source data

To control for potential artifacts from tissue processing, we also isolated EVs secreted into the culture medium of a human embryonic kidney (HEK) cell model of tau assembly. In this model, the assembly of expressed full-length P301S 1N4R tau fused to yellow fluorescent protein (YFP) was seeded using tau filaments extracted from the brains of individuals with AD (Extended Data Table 1), and clonal cell lines that stably propagate assembled tau were selected (Extended Data Fig. 4a). NTA of these medium-derived EVs showed a similar size profile to the EVs from the brains of individuals with AD, including the presence of large EVs >300 nm in diameter (Extended Data Fig. 4b). In agreement with our data from the human brain, the EVs were associated with assembled truncated tau and were enriched in known EV and endo-lysosomal proteins (Extended Data Fig. 4c).

### EV-associated tau is hyperphosphorylated and assembled

**Extended Data Fig. 5:**
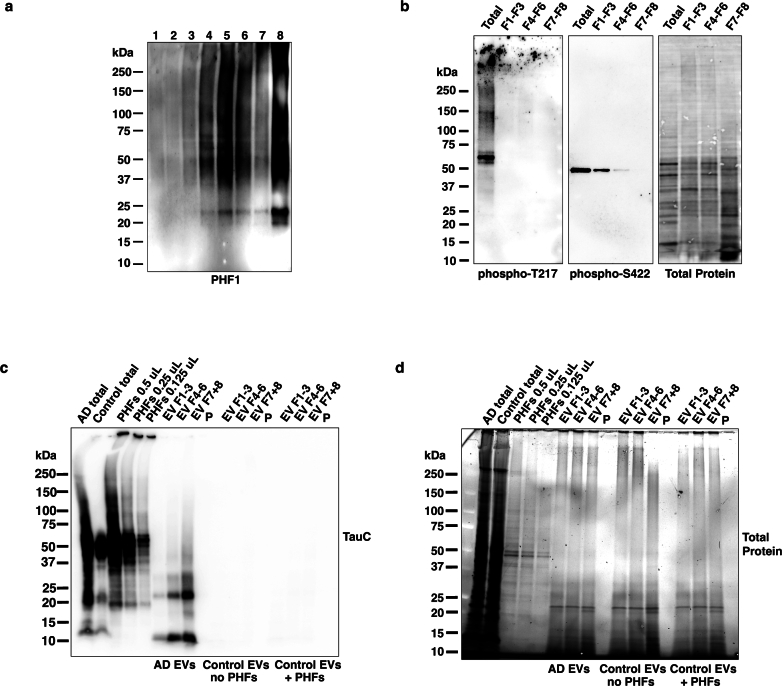
Characterisation and co-purification controls of human brain derived EVs. **a**, Representative immunoblots of EV fractions 1–8 isolated from AD brain using antibody PHF1 (phospho-S396 and -S404 tau). n = 3. **b**, Representative immunoblots of EV fractions 1–3, 4–6 and 7-8 isolated from AD brain using antibodies against phospho-T217 and phospho-S422 tau. n = 3. **c,d**, Representative immunoblot using antibody TauC (tau repeat region; **c**) and corresponding stain-free total protein loading control (**d**) of AD and control brain homogenates (total); PHFs extracted from AD brain; pooled EV fractions 1–3, 4–6, and 7-8 from AD and control brain, with and without spiked PHFs extracted from AD brain; and the pellet following EV fractionation (P). n = 2. Source data

Immunoblotting using PHF1 (anti-phospho-S396 and phospho-S404 tau) showed that hyperphosphorylated tau was present in EV fractions 3–8 from the brains of individuals with AD (Extended Data Fig. 5a). The 12-kDa tau fragment was not detected by PHF1, consistent with the C-terminal truncation of this fragment. The 12- and 24-kDa tau fragments were not detected by antibodies to phospho-T217 and phospho-S422 tau (Extended Data Fig. 5b), in agreement with N- and C-terminal truncation.

Incubation of the EV fractions with the detergent sarkosyl, followed by differential centrifugation and immunoblotting, demonstrated that the truncated tau was sarkosyl insoluble and, therefore, assembled (Fig. 2d)^1^. To test for contamination by cell-derived sarkosyl-insoluble tau, we spiked brain tissue from individuals without tau pathology with the sarkosyl-insoluble fraction of AD brain tissue during tissue dissociation and proceeded to isolate EVs (Extended Data Fig. 5c,d). Sarkosyl-insoluble tau did not coisolate with EVs, suggesting that cell-derived sarkosyl-insoluble tau was not contaminating our EV samples and was not incorporated into vesicles during the EV isolation protocol. These results show that hyperphosphorylated, assembled truncated tau containing the repeat region is associated with EVs from the brains of individuals with AD.

### EVs from the brains of individuals with AD contain tau PHFs and SFs

**Extended Data Fig. 6:**
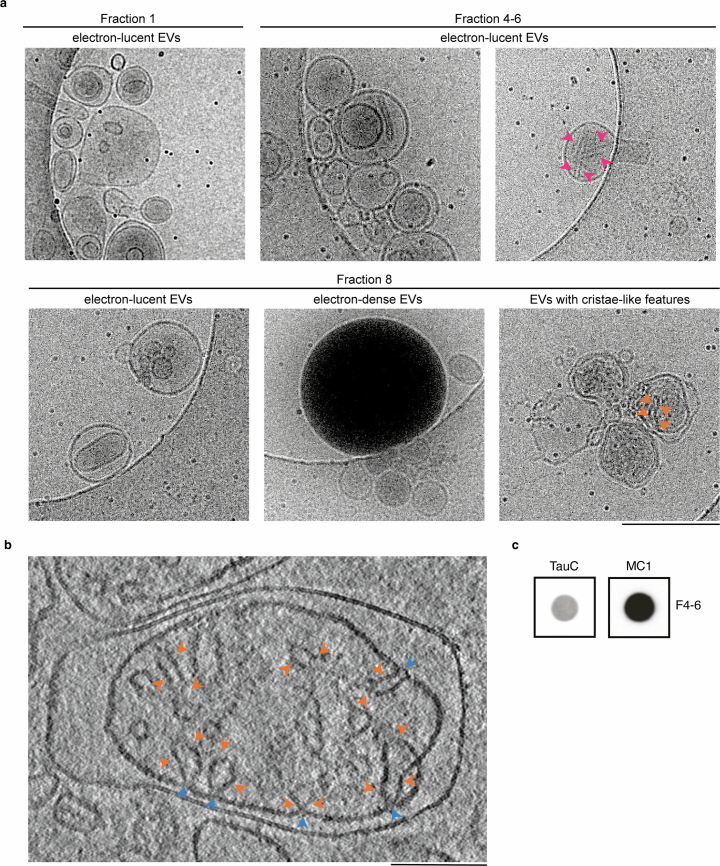
Cryo-EM of extracellular vesicles isolated from Alzheimer’s disease patient brain. **a**, Cryo-EM images of morphologically-distinct EV populations in fraction 1, pooled fractions 4–6 and fraction 8 from an individual with AD. Electron-lucent EVs were observed in all fractions. Electron-dense EVs and electron-lucent EVs with cristae-like features were only observed in fraction 8. EVs associated with tau filaments were only observed in pooled fractions 4–6. Magenta arrows indicate examples of PHF-like filaments and orange arrows indicate examples of cristae-like features. Scalebar, 500 nm. **b**, Deconvoluted tomographic slice of an EV from fraction 8 with cristae-like features. Orange arrows indicate cristae-like structures and blue arrows indicate membrane invaginations at the necks of cristae-like structures. Scalebar, 200 nm. **c**, Representative dot blots of the sarkosyl-insoluble fraction of pooled EV fractions 4–6 from AD patient brain using antibodies TauC and MC1.

We used cryo-EM to image intact EVs from fraction 1, pooled fractions 4–6 and fraction 8 under near-native conditions. EVs in fractions 1 and pooled fractions 4–6 were electron lucent, whereas fraction 8 comprised both electron-lucent and electron-dense EVs (Extended Data Fig. 6). A subset of the electron-lucent EVs in fraction 8 contained cristae-like membrane structures (Extended Data Fig. 6a,b), consistent with their enrichment in mitochondrial proteins (Fig. 1a,c). We observed electron-lucent EVs in fractions 4–6 that were associated with helical filaments that resembled tau PHFs and SFs by their dimensions and helical crossover spacings (Extended Data Fig. 6a). EVs associated with tau-like filaments were not observed in fractions 1 and 8. Manual inspection and counting of EVs from fractions 4–6 on cryo-EM grids indicated that approximately 1 in 1,000 EVs were associated with tau filaments. Dot blots of the sarkosyl-insoluble material from EV fractions 4–6 using the conformational antibody MC1 supported the presence of tau PHFs and SFs^38^ (Extended Data Fig. 6c).

**Fig. 3:**
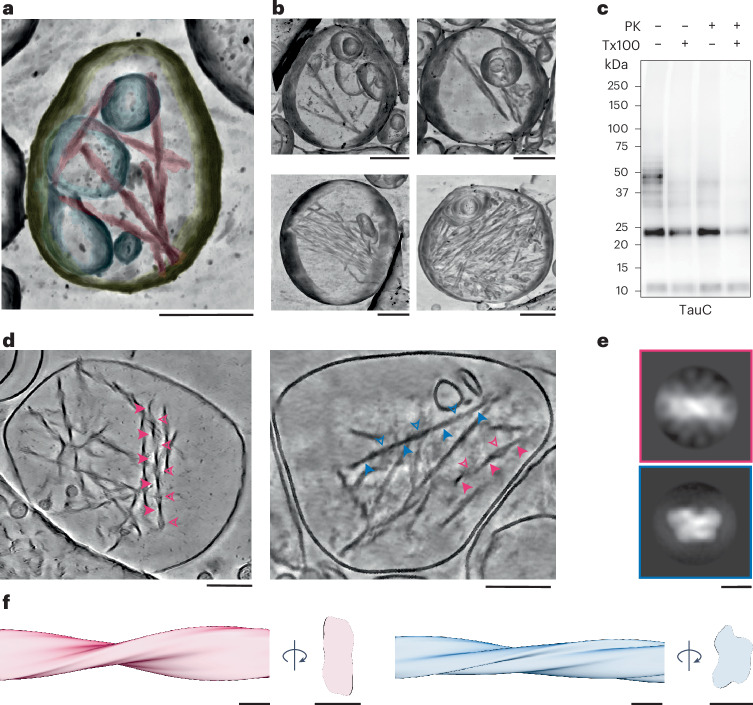
Cryo-ET of EVs containing tau PHFs and SFs from the brains of individuals with AD. **a**, Denoised tomographic volume and segmentation of an EV from the brain of an individual with AD depicting the limiting membrane (yellow), internal vesicles (cyan) and tau filaments (magenta). **b**, Denoised tomographic volumes of EVs from the brains of individuals with AD containing varying numbers of tau filaments. **c**, Representative immunoblot of EVs from the brains of individuals with AD from fractions 4–6 using antibody TauC with (+) or without (−) 0.1% Triton X-100 (Tx100) and 0.5 ng µl^–1^ proteinase K (PK); *n* = 3. **d**, Denoised tomographic slices of EVs from the brains of individuals with AD containing tau filaments. Arrows point to the minimum (filled arrows) and maximum (unfilled arrows) widths of tau PHFs (magenta arrows) and SFs (blue arrows). **e**,**f**, Subtomogram averaged maps of tau PHFs (magenta) and SFs (blue) in EVs, shown as central slices perpendicular to the helical filament axis (**e**) and three-dimensional (3D) volumes encompassing one helical crossover (**f**). See also Extended Data Fig. 7c,d; scale bars, 200 nm (**a**, **b** and **d**) and 10 nm (**e** and **f**). Source data

**Extended Data Fig. 7:**
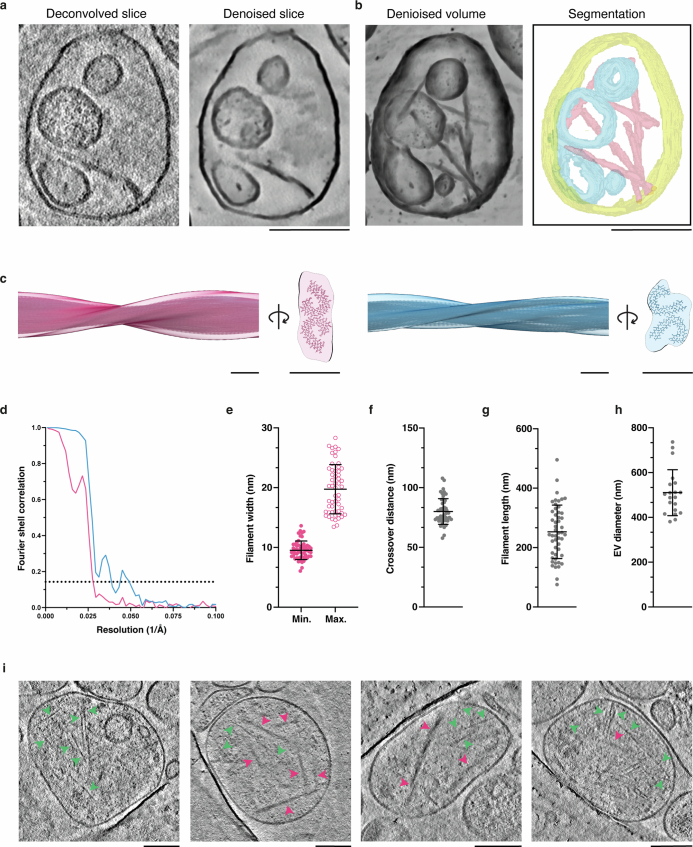
Cryo-ET of extracellular vesicles containing tau PHFs and SFs from Alzheimer’s disease patient brain continued. **a**, Deconvolved and denoised tomographic slices of the EV containing tau PHFs and SFs from AD brain shown in Fig. 3a. **b**, Denoised tomographic volume and segmentation of the EV containing tau PHFs and SFs from AD brain shown in Fig. 3a. The limiting membrane is highlighted in yellow, intraluminal vesicles in cyan, and PHFs in magenta. **c**, Fit of atomic models (grey) of PHFs (PDB ID 6HRE) and SFs (PDB ID 7nrs) to the subtomogram averaged maps of tau PHFs (magenta) and SFs (blue) in EVs. **d**, Fourier shell correlation (FSC) curves for the two independently refined subtomogram averaged half-maps of the PHF-like filament type (magenta line) and the SF-like filament type (blue line). The FSC of 0.143 is shown with a dashed line. **e**, Measurements of the minimum and maximum widths of tau filaments in EVs from AD patient brain. n = 55. **f**, Measurements of the helical crossover distance (the distance between minimum and maximum widths) of tau filaments in EVs from AD patient brain. n = 50. **g**, Measurements of the lengths of tau filaments in EVs from AD patient brain. n = 50. **h**, Measurements of the diameters of EVs containing tau filaments from AD patient brain. n, 20. **i**, Deconvolved tomographic slices of EVs from AD brain containing tau filaments (magenta arrows) and non-tau filaments (green arrows). **j**, Deconvolved tomographic slices of Sarkosyl-extracted tau filaments from EVs, rarely decorated by additional densities. Orange arrows indicate additional densities that decorate tau filaments and cyan arrows indicate densities that are proximal to, but not associated with, tau filaments. Scale bars, 200 nm in (a–c, i and j) and 10 nm in (c).

We used cryo-ET to reconstruct molecular-resolution tomograms of the EVs associated with tau filaments. This revealed that the filaments were contained within the lumen of the EVs, which had intact limiting membranes (Fig. 3a and Extended Data Fig. 7a,b). Variable numbers of filaments were observed per EV, ranging from 3 to more than 50 (Fig. 3b). A protease protection assay supported the observation that tau filaments were contained within the lumen of intact EVs. Tau associated with EVs could only be degraded by proteinase K following treatment with detergent to permeabilize EV membranes (Fig. 3c).

Subtomogram averaging of the filaments within EVs yielded two filament types (Fig. 3d–f and Extended Data Fig. 7c,d). The first type resembled tau PHFs, which have a C2 symmetrical, base-to-base packing of the C-shaped protofilaments^1^. The second type resembled tau SFs, in which the protofilaments pack in a nonsymmetrical, back-to-back arrangement. This analysis was supported by measurements of single filaments. Tau filaments had average helical crossovers of about 80 nm and cross-sectional dimensions of between 7 and 20 nm (Extended Data Fig. 7e,f), in agreement with the dimensions of tau PHFs and SFs^1,2^. The filaments were between 75 and 500 nm in length (Extended Data Fig. 7g). This is shorter than tau filaments observed within neurofibrillary tangles, which span micrometers^39^. We also observed filaments in EVs that were morphologically distinct from PHFs and SFs (Extended Data Fig. 7i). These filaments matched the dimensions of intermediate filaments and F-actin, both of which have previously been reported in EVs^35,40^.

The EVs containing tau filaments had single limiting membranes and often contained internal vesicles (Fig. 3a,b,d). Both the EVs and the internal vesicles displayed variable granularity. The EVs were large compared to the majority of EVs, with diameters of >300 nm (Extended Data Fig. 7h). These observations were supported by our NTA measurements, which showed that approximately 6% of EVs had diameters of >300 nm (Extended Data Fig. 2e–h). These observations are consistent with previous reports of large EVs that contain internal vesicles from cell cultures and tissues^35,41,42^. Our results show that short tau PHFs and SFs are contained within the lumen of EVs from the brains of individuals with AD.

### Tau filaments are tethered to the EV limiting membrane

**Fig. 4:**
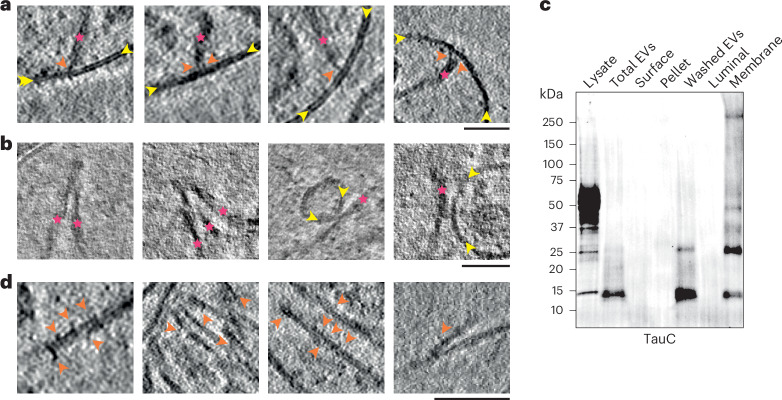
Tau filaments are tethered to the limiting membrane of EVs from the brains of individuals with AD and are decorated by additional densities. **a**,**b**, Deconvolved tomographic slices of EVs from the brains of individuals with AD showing tethering of the ends of tau filaments to the luminal side of the limiting membrane (**a**) and lateral filament contacts between filaments and with internal vesicles (**b**). The magenta asterisks indicate filaments, the yellow arrows point to the limiting membranes of EVs (**a**) and internal vesicles (**b**), and the orange arrows point to densities tethering the ends of filaments to the luminal side of the limiting membrane. **c**, Representative immunoblot using antibody TauC showing total EVs, proteins liberated from the EV surface following a dithiothreitol (DTT) wash (Surface), sedimented material (Pellet; not associated with membranes), DTT-washed EVs (Washed EVs), luminal proteins following resuspension of DTT-washed EVs in hypotonic buffer (Luminal) and the sodium carbonate-treated membrane-associated fraction (Membrane); *n* = 3. **d**, Deconvolved tomographic slices of EVs from the brains of individuals with AD showing additional densities decorating the lengths of tau filaments. Orange arrows point to additional densities; scale bars in **a**, **b** and **d** indicate 200 nm. Source data

Cryo-ET revealed that at least one tau filament per EV was tethered to the luminal side of the EV limiting membrane (Fig. 4a). Tethering to the EV limiting membrane occurred exclusively at filament ends and was mediated by flexible elongated densities that often appeared to traverse the limiting membrane.

The remaining filaments made lateral contacts with the tethered filament and/or with one another (Fig. 4b), resulting in a network of filaments originating from the site of tethering (Fig. 3a,b,d). We did not observe free-floating filaments in EVs. Occasionally, the sides of filaments also made contact with internal vesicles (Fig. 4b). These lateral contacts between filaments and between filaments and internal vesicles were distinct from the tethers linking filament ends to the EV limiting membrane. Whereas the tethers are well-defined densities, the lateral contacts appear fuzzy and may represent direct contact points.

Fractionation of EVs into surface proteins, luminal proteins and membrane-associated proteins supported the observation that the filaments associated with one another and were tethered to the limiting membrane. Tau was retained in the membrane fraction following treatment of EVs with 0.1 M sodium carbonate (pH 11), suggesting membrane interaction^43^ (Fig. 4c). These results show that tau filaments within EVs in the brains of individuals with AD associate with one another and are tethered to the limiting membrane.

In addition, we observed globular densities that decorated the sides of the filaments (Fig. 4d). These globular densities were rarely observed in tomograms of tau filaments extracted from EVs from the brains of individuals with AD using the detergent sarkosyl (Extended Data Fig. 7j), suggesting that they are lost during the extraction procedure.

### Tau PHFs within EVs contain additional molecules

**Fig. 5:**
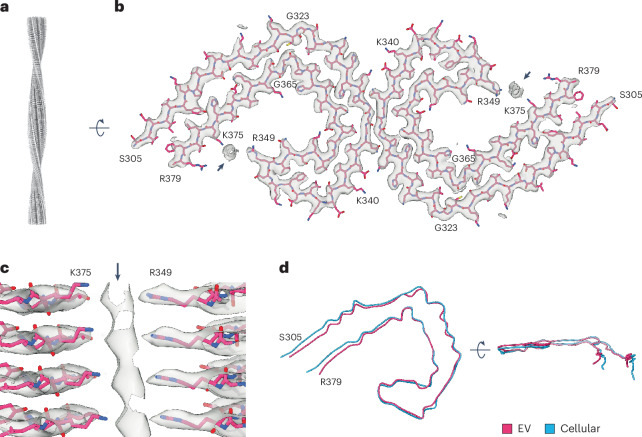
Cryo-EM structure of EV-derived tau PHFs from the brains of individuals with AD. **a**, Cryo-EM map of tau PHFs from EVs derived from the brain tissue of individuals with AD viewed aligned to the helical axis. See also Extended Data Fig. 8a–g. **b**, Cryo-EM map (transparent gray) and atomic model (magenta) of tau PHFs from EVs derived from the brain tissue of individuals with AD, shown for a single tau molecule per protofilament perpendicular to the helical axis. Additional densities coordinated to the side chains of R349 and K375 are indicated with arrows. **c**, Magnified view of the cryo-EM map (transparent gray) and atomic model (magenta) showing the additional density, indicated with an arrow, coordinated to the side chains of R349 and K375. Four tau molecules are depicted aligned to the helical axis. **d**, Overlay of the tau filament folds from EVs (magenta) and the cellular fraction (cyan) from the brains of individuals with AD. See also Extended Data Fig. 8h.

**Extended Data Fig. 8:**
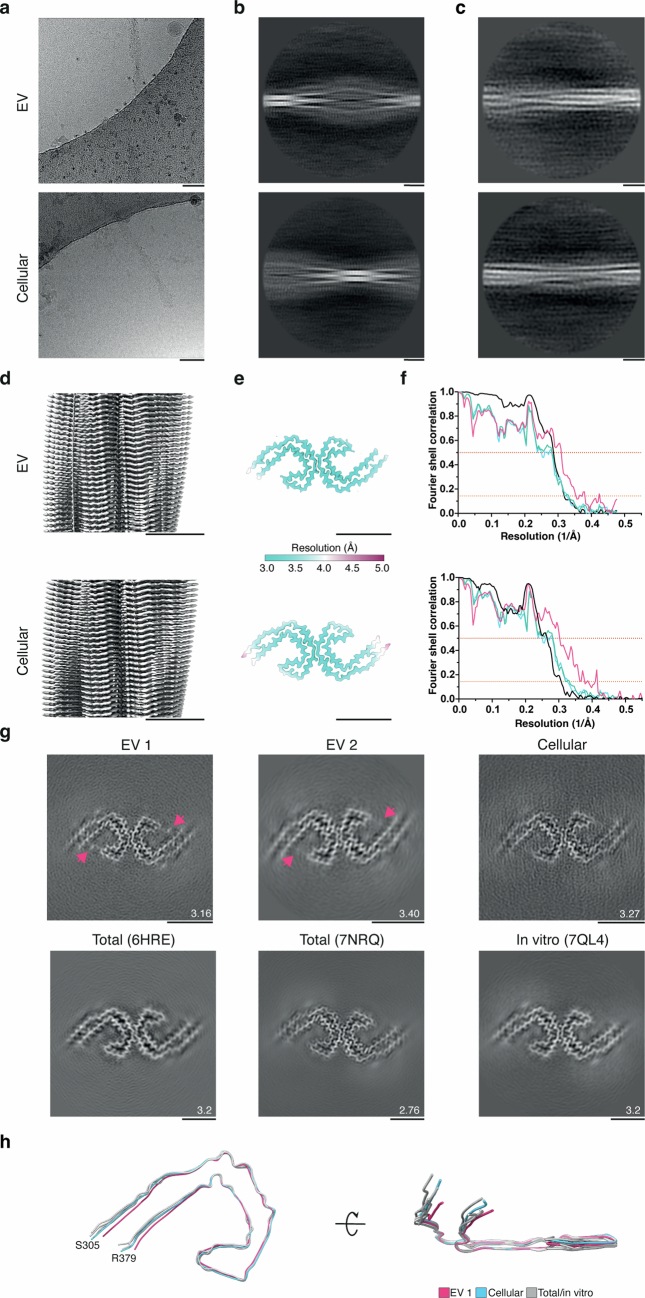
Comparison of cryo-EM structures of PHFs from EV and cellular fractions of Alzheimer’s disease brain. **a**, Representative cryo-EM images of tau filaments extracted from EVs and from the cellular fraction from AD patient brain. **b**, Representative reference-free two-dimensional (2D) cryo-EM class averages of PHFs from EVs from AD patient brain. **c**, Representative reference-free 2D cryo-EM class averages of SFs from EVs from AD patient brain. **d**, Cryo-EM maps viewed aligned to the helical axis of PHFs extracted from EVs (top) and from the cellular fraction (bottom) from AD brain. **e**, Local resolution estimates for the cryo-EM maps of PHFs extracted from EVs (top) and from the cellular fraction (bottom) from AD brain. **f**, Fourier shell correlation (FSC) curves for the two independently refined cryo-EM half-maps (black line); for the refined atomic model against the cryo-EM density map (magenta); for the atomic model shaken and refined using the first half-map against the first half-map (teal); and for the same atomic model against the second half-map (cyan) of PHFs extracted from EVs (top) and from the cellular fraction (bottom) from AD brain. The FSC thresholds of 0.143 and 0.5 are shown with orange and red dashed lines, respectively. **g**, Cryo-EM maps of PHFs from EVs from two different individuals, the cellular fraction from one individual, total brain homogenate from two different individuals (PDB IDs 6HRE and 7NRQ), and in vitro reconstitution (PDB ID 7QL4), shown as central slices perpendicular to the helical axis. Additional densities in the structures of PHFs from EVs are indicated with arrows. The resolutions (in Å) of the maps are given in the bottom right-hand corner of the images. **h**, Overlay of the PHF folds from EVs (magenta), the cellular fraction (cyan), and total brain homogenate and in vitro reconstitution (PDB IDs 6HRE, 7NRQ and 7QL4) (grey). **a**-**e** and **g**, Scale bars, 50 Å.

We used single-particle cryo-EM to analyze tau filaments extracted from EVs isolated from the brains of two individuals with AD (Extended Data Fig. 8a). Reference-free two-dimensional (2D) class averaging confirmed the presence of tau PHFs and less-abundant SFs (Extended Data Fig. 8b,c), which comprised approximately 88% and 12% of filament segments, respectively. This is similar to the ratios of PHFs to SFs previously reported in total brain homogenates^2^. We determined the cryo-EM structure of the tau PHFs, which revealed that the filaments had the same C-shaped protofilament fold formed by residues S305–R379 as those from total brain homogenates from individuals with AD^1,2^ (Fig. 5a,b and Extended Data Fig. 8d–f). Two C-shaped protofilaments were related by helical 2–1 screw symmetry, with a protofilament interface formed by residues K331–E338. Due to their low abundance, it was not possible to determine the cryo-EM structure of the SFs. However, we note that PHFs and SFs have identical protofilament folds in cryo-EM structures from total brain homogenates^1,2^.

Unlike PHFs isolated from total brain homogenates and PHFs reconstituted in vitro from a fragment of tau comprising residues 297–391 (refs. ^1,2,44^), the PHFs from EVs contained additional densities between the positively charged side chains of R349 and K375, revealing the presence of anionic molecules (Fig. 5b,c and Extended Data Fig. 8g). Their identity was not resolved, possibly because they do not share the helical symmetry of the tau molecules. These additional molecules were accompanied by a more compacted C-shaped filament fold than that of PHFs from total brain homogenates and of PHFs reconstituted in vitro (Fig. 5d and Extended Data Fig. 8h). Compaction was mediated by hinging of the main chain at G323 and G366 in the cross-β region and at K340 and I354 in the β-helix region.

We also determined the structure of PHFs extracted from the cellular brain fraction from one of the individuals with AD (Fig. 5d and Extended Data Fig. 8). This was identical to those determined from total homogenates and reconstituted in vitro^1,2,44^. We conclude that PHFs within brain EVs from the brains of individuals with AD contain additional anionic molecules compared to those within neurofibrillary tangles.

## Discussion

The association of assembled tau with EVs in the central nervous system of individuals with AD has been linked to its clearance and prion-like propagation^22–25^. However, our understanding of the molecular mechanisms underlying EV-mediated secretion of assembled tau is limited because the molecular species of assembled tau, the identities of the EVs that associate with assembled tau and how tau associates with EVs have not been determined.

We performed a comprehensive density-based profiling of brain EVs from individuals with AD. This revealed the enrichment of proteins from distinct intracellular compartments of EV biogenesis in different EV density fractions, including from the plasma membrane, endo-lysosomes and mitochondria. We found that EVs containing tau filaments were enriched in endo-lysosomal proteins. Association of lysosomal substrates with EVs has been observed as a consequence of lysosomal impairment^45–48^, including assembled protein substrates^48,49^. This suggests that tau filaments may be secreted in EVs as a result of impaired lysosomal degradation. Tau filaments are targeted to lysosomes in cell and mouse models^50^. Lysosomal impairment has been described in AD, and multiple genetic risk factors for AD are implicated in lysosomal dysfunction^50^. We also identified enrichment of astrocyte, oligodendrocyte and neuronal marker proteins across EV fractions. In addition to EVs released by neurons, glial cell EVs may play important roles in the clearance and prion-like propagation of assembled tau^51^. A limitation of this approach is that it is not able to identify if there is differential protein enrichment among EVs within individual fractions, which will require technologies that allow for protein profiling of single EVs.

Potential artifacts due to membrane damage need to be considered when isolating EVs from tissue samples^31^. To minimize membrane damage, we used fresh-frozen postmortem brain tissue with the best possible preservation and avoided mechanical tissue disruption (see Methods). We used multiple orthogonal approaches to test for the presence of artifacts. Quantitative mass spectrometry and immunoblotting showed enrichment of EV-associated proteins and depletion of common protein contaminants^31^ (Fig. 1 and Extended Data Fig. 1). Cryo-EM imaging demonstrated that the EVs had intact membranes and were not contaminated by intracellular components (Extended Data Fig. 6a). To control for potential artifacts due to membrane damage, we analyzed EVs secreted into the culture medium of a cell model of tau assembly, which were also associated with assembled truncated tau and had a similar size profile as the EVs from the brains of individuals with AD (Extended Data Fig. 4). We also spiked control brain tissue with exogenous tau filaments and observed that these were not incorporated into vesicles during the EV isolation protocol (Extended Data Fig. 5c). We conclude that artifacts due to membrane damage are unlikely to have influenced the results of our study.

Although immunoblotting showed that EV fraction 8 also contained hyperphosphorylated, truncated insoluble tau (Fig. 2c,d and Extended Data Fig. 5a), we did not observe tau filaments within EVs from this fraction (Extended Data Fig. 6a). It could be that tau is contained within the electron-dense EVs unique to fraction 8 (Extended Data Fig. 6a), as these are too electron dense to resolve using cryo-ET or other electron microscopy approaches. New techniques will be required to investigate this possibility.

The relatively low abundance of EVs containing tau filaments (1 in 1,000) is consistent with our observation that tau filaments were confined to EVs with large diameters, which made up only approximately 6% of total EVs. A relatively low abundance of EVs containing tau filaments is expected given the diverse roles performed by EVs, with distinct cell types each releasing multiple types of EVs, and the fact that not all brain cells exhibit tau pathology. It has been estimated that only 0.5–11.7% of neurons exhibit neurofibrillary tangles at end-stage AD^52^. In addition to neuronal EVs, our samples also contained EVs from other brain cell types (Extended Data Fig. 3).

It was not known which molecular species and structures of assembled tau associate with EVs. We showed that PHFs and SFs composed of tau largely truncated at the N and C termini are found within the lumen of EVs in the brains of individuals with AD. Truncation is compatible with the cryo-EM structure of the C-shaped protofilament fold, which encompasses residues 305–379. The enrichment of peptides encompassing tau residues 6–23 (Fig. 2b) is supported by the immunoreactivity of the tau filaments against the antibody MC1 (Extended Data Fig. 6c), which detects a discontinuous conformational epitope of filamentous tau comprising tau residues 7–9 and 313–322 (ref. ^38^). This suggests that these N-terminal peptides remain associated with tau filaments following truncation of the intermediate tau residues. Similarly, the MC1 immunoreactivity of tau filaments in Pick’s disease was also preserved following their truncation by pronase^53^. Filaments of truncated tau have previously been observed in total brain tissue homogenates from individuals with AD and have been shown to seed tau assembly^54,55^. Our results suggest that tau proteolysis might occur within the protease-rich environment of EVs or in the endo-lysosomal pathway before EV biogenesis. Our finding that tau is truncated and that the residues S305–R379 are buried in the ordered filament core will inform the development of tools that target assembled EV-associated tau for therapeutic and biomarker applications.

Tau PHFs and SFs within EVs were short compared to those in neurofibrillary tangles. Similar short tau filaments generated by homogenization and fractionation of human or transgenic mouse brain have been shown to have the greatest ability to seed tau assembly in model systems^14,56–58^. This study represents the first observation of short tau filaments in situ in a human brain environment. Our results support the hypothesis that short tau filaments may act as seeds for prion-like propagation in the human brain.

We observed additional anionic molecules in PHFs from EVs compared to PHFs from the cellular fraction, demonstrating that tau filament composition can differ between cellular compartments. The anionic molecules may be derived from the EV lumen or from environments upstream of EV biogenesis. Using cryo-ET, we also observed additional globular densities that decorated the sides of the filaments. Such densities were rarely observed in tomograms and micrographs of sarkosyl-extracted tau filaments from the brains of individuals with AD (Extended Data Figs. 7j and 8a)^1,2^, suggesting that these additional densities are lost during the extraction procedure. Co-immunoprecipitation experiments have shown that a large number of proteins interact with tau filaments in the human brain^58^. The identities of the molecules that interact with tau filaments in EVs, as well as if they influence filament formation and EV-mediated secretion, remain to be determined. Future studies should use cryo-ET to examine EVs from different brain regions and from additional individuals with AD.

How assembled tau associates with EVs was unknown. Our results reveal that tau filaments are contained within the lumen of EVs. This finding has important implications for therapeutic approaches targeting extracellular tau, which will be inaccessible to antibodies and membrane-impermeable compounds. Within the lumen of EVs, at least one tau filament was tethered to the limiting membrane (Fig. 4a). The remaining tau filaments made lateral contacts with the tethered filament(s) and with one another so that there were no free-floating filaments in the EV lumen (Fig. 4b). This was supported by recovery of the tau filaments in the membrane-associated fraction following EV fractionation (Fig. 4c). The lateral contact sites appeared fuzzy and were distinct from the well-defined densities that tethered the ends of tau filaments to the EV limiting membrane. The presence of such densities at membrane tethering sites suggests that the filaments may be actively sorted into EVs. Tethering may require adaptor molecules, such as BIN1 (ref. ^24^), or may be mediated by direct polypeptide anchoring to membranes^59^. That filaments were uniquely tethered at their ends suggests that sequences or structural motifs exposed at the filament ends are important for binding to the tethers. Targeting of the tethering mechanism may represent a strategy to modulate EV-mediated secretion of tau filaments.

We have shown that PHFs and SFs of truncated tau are tethered within EVs enriched in endo-lysosomal proteins in the brains of individuals with AD. This work paves the way for further in situ cryo-ET studies of neurodegenerative disease-associated protein assemblies in human brain environments. Our results will guide mechanistic studies into the secretion of tau filaments within EVs as well as inform strategies to target extracellular tau.

## Methods

### Statistics and reproducibility

No statistical methods were used to predetermine sample sizes for the number of human samples used; however, sample sizes are the same or larger than those used in comparable studies. We consistently observed a similar distribution of tau and reported EV protein markers across all eight human donors and eight EV density fractions using mass spectrometry. As described in the Methods, three EV density fraction 8 samples (from donors A, F and E) were removed from the mass spectrometry analysis, as significantly less material was isolated from those donors. Data collection and analysis were not performed blind, as all specimens observed were from donors diagnosed with AD. Data distribution was assumed to be normal, but this was not formally tested. The statistical analyses performed for each experiment are listed in the associated figure legends. Statistical analyses were performed using GraphPad Prism.

### Isolation of EVs from postmortem human brain tissue

Human tissue samples were sourced from the New York Brain Bank at Columbia University (Alzheimer’s Disease Research Center) and the University of Miami Brain Endowment Bank. Their use in this study was approved by the ethical review processes at each institution. Informed consent was obtained from the individuals’ next of kin. EVs were isolated essentially as previously described^36^, with minor alterations to achieve a gentler tissue dissociation suitable for fresh-frozen postmortem human brain tissue. Tissue samples had low or no time to cold (~1.5 h average) and low overall frozen postmortem intervals (~13.5 h average; Extended Data Table 1). Tissue samples were dissected and frozen from fresh using liquid nitrogen vapor to minimize freezing artifacts^60^. Frozen tissue (0.8–1.2 g) was transferred directly to Petri dishes to minimize thawing artifacts^61^ and gently sliced into pieces (1–5 mm^3^) using a razor blade in a few drops of prewarmed (37 °C) Hibernate A medium containing 20 U ml^–1^ papain (Worthington, LK003178). We avoided mincing, vortexing or homogenization, which can damage membranes^62^. Sliced tissue was incubated in 3.125 ml of prewarmed papain solution for 10 min at 37 °C, with three gentle tube inversions after 5 min. Papain digestion was stopped by the addition of 6.5 ml of ice-cold Hibernate A medium containing protease inhibitors (5 μg ml^–1^ leupeptin, 5 μg ml^–1^ antipain dihydrochloride, 5 μg ml^–1^ pepstatin A, 1 mM phenylmethanesulfonyl fluoride and 1 μM E64), and the dissociated tissue was passed slowly through a wide-bore 10-ml pipette until smooth (six to eight times). The dissociated tissue was then centrifuged at 300*g* for 10 min at 4 °C to pellet cells. The supernatant, containing the interstitial fluid, was passed through a 40-μm cell strainer (Corning, 352340), followed by a low-protein-binding 0.2-μm syringe filter (Corning, 431219). The filtrate was centrifuged at 2,000*g* for 10 min at 4 °C, and the supernatant was made to 60 ml with ice-cold PBS (Gibco, 10010-015) and further centrifuged at 10,000*g* for 30 min at 4 °C. The supernatant was transferred to fresh tubes and centrifuged at 100,000*g* for 70 min at 4 °C using a Ti45 rotor (Beckman). The 100,000*g* pellets were resuspended in 1 ml of ice-cold PBS and brought to 60 ml using PBS before another round of centrifugation at 100,000*g* for 70 min at 4 °C. The supernatants were discarded, and the pellets (containing EVs) were resuspended in 1.5 ml of 60 mM Tris-HCl (pH 7.4) containing 0.25 M sucrose and 40% Optiprep (OP) and placed in ultraclear centrifuge tubes (Beckman, 344059). Discontinuous OP gradients were prepared by layering 2 ml of 60 mM Tris-HCl (pH 7.4) containing 0.25 M sucrose and 20, 15, 13, 11, 9, 7 and 5% OP, respectively. Additional 60 mM Tris-HCl (pH 7.4) containing 0.25 M sucrose and 5% OP was then added until 1–3 mm from the top of the tube. The prepared gradients were centrifuged at 200,000*g* for 16 h at 4 °C in an SW41Ti rotor (Beckman) with no braking during deceleration. Eight fractions encompassing each OP concentration were collected into 30-ml thickwall polycarbonate tubes (Beckman, 355631), as described in Supplementary Fig. 1 (fraction 1 = 5% OP; fraction 2 = 7% OP; fraction 3 = 9% OP; fraction 4 = 11% OP; fraction 5 = 13% OP; fraction 6 = 15% OP; fraction 7 = 20% OP; fraction 8 = interface between the 20/40% OP layers). Fractions were diluted with 15 ml of cold PBS and centrifuged at 100,000*g* for 70 min at 4 °C using a 70.1Ti rotor (Beckman). The supernatants were discarded, and the pellets (containing EVs) were resuspended in 50 μl of PBS. EV suspensions were used immediately for cryo-ET and for proteinase K protection assays, or aliquoted and frozen at −80 °C for additional biochemical analyses, including before sarkosyl extraction for cryo-EM.

### Immunoblots

EV suspensions in PBS were diluted 1:1 in 2× RIPA (100 mM Tris-HCl (pH 7.4), 300 mM NaCl, 2% NP-40, 1% sodium deoxycholate and 0.2% SDS). Total protein concentration was determined using a bicinchoninic acid assay. EV suspensions were normalized to total protein concentration, prepared with 4× Laemmli sample buffer (Bio-Rad, 1610747) containing 50 mM DTT and heated at 95 °C for 5 min. Samples were resolved using 4–20% Tris-glycine extended stain-free gels (Bio-Rad). Proteins were transferred to low-fluorescence PVDF membranes (Bio-Rad) and visualized using UV light in a ChemiDoc MP imager (Bio-Rad). Membranes were subsequently blocked with 5% milk powder in Tris-buffered saline (20 mM Tris-HCl (pH 7.4) and 150 mM NaCl) containing 0.02% Tween 20 (TBST) for 1 h at 21 °C. Primary antibodies were diluted in Superblock TBS blocking buffer (Thermo Fisher Scientific) and incubated overnight with the membranes. Membranes were then washed twice in TBST and twice in TBST containing 5% milk powder, with each wash lasting 10 min. Secondary horseradish peroxidase (HRP)-conjugated antibodies were diluted in TBST containing 5% milk powder and incubated with the membranes for 1 h at 21 °C. Membranes were then washed four times in TBST for 10 min each wash, and labeled proteins were visualized using Bio-Rad Clarity Max chemiluminescent substrate in a ChemiDoc MP imager. The total protein signal visualized using UV light was used to normalize the labeled protein signal using the Image Lab software package, version 6.1, build 7 (Bio-Rad). The following primary antibodies were used: annexin A2 (Cell Signaling, D11G2; 1:1,000), flotillin 1 (BD Transduction, 610821; 1:1,000), CD81 (Cell Signaling, D3N2D; 1:1,000), LAMP1 (Abcam, ab62562; 1:250), LAMP2 (Santa Cruz, sc-18822, clone H4B4; 1:500), VDAC (Cell Signaling, 4866; 1:1,000), lamin A/C (Santa Cruz, sc-376248; 1:1,000), Tau13 (BioVision, 3453-100; 1:1,000), HT7 (Invitrogen, MN1000; 1:1,000), TauC (Dako, A0024; 1:5,000), PHF1 (mouse monoclonal; gift from P. Davies; 1:250), EEA1 (BD Biosciences, 610457; 1:250), β-actin–FITC (Sigma-Aldrich, F3022; 1:1,000), MC1 (gift from P. Davies; 1:250), phospho-tau-T217 (Thermo Fisher Scientific, PA5-37639; 1:250) and phospho-tau-S422 (Thermo Fisher Scientific, 44-764G; 1:250).

### Dot blots

Sarkosyl-insoluble extracts from pooled EV fractions 4–6 as well as sarkosyl-insoluble extracts from total brain homogenates were transferred onto nitrocellulose membranes (0.2-µm pore size; Bio-Rad, 162-0112) using the 96-well Minifold I Dot-Blot System (Whatmann). The membranes were incubated in a blocking buffer containing PBS supplemented with 0.1% Tween 20 (PBST) and 1% bovine serum albumin (BSA) for 30 min at 21 °C, followed by incubation in blocking buffer containing the antibody MC1 (1:250) overnight at 4 °C. The membranes were washed three times in PBST for 10 min each wash and incubated in HRP-conjugated secondary antibodies (Bio-Rad; 1:5,000) for 1 h at 21 °C. After washing three times in PBST for 10 min each wash, the membranes were incubated with Enhanced Chemiluminescence Prime reagents (Amersham) for 1 min at 21 °C and imaged using a ChemiDoc MP (Bio-Rad). The membranes were washed three times in PBST for 10 min each wash and incubated in blocking buffer for 30 min at 21 °C before incubation in blocking buffer containing the antibody TauC (Dako, A0024; 1:5,000) for 1 h at 21 °C. After washing the membrane three times in PBST for 10 min each wash, the membranes were incubated in DyLight 800-conjugated secondary antibodies (Cell Signaling; 1:2,500) for 1 h at 21 °C. The membranes were washed three times (10 min each) in PBST and imaged using a ChemiDoc MP (Bio-Rad).

### Proteomic sample preparation and liquid chromatography–tandem mass spectrometry analysis

#### Reduction, alkylation, S-TRAP clean-up and protease digestion

EV fractions were resuspended in TBS (20 mM Tris-HCl (pH 7.4) and 150 mM NaCl) containing 5% SDS, and proteins were digested using S-TRAP Micro spin columns, according to the manufacturer’s protocol (Profiti). Approximately 50 μl of starting material was used for each experiment, corresponding to ~50 μg of total protein. Samples were reduced by the addition of 20 mM DTT and incubated for 30 min at 21 °C. Samples were alkylated by the addition of 40 mM iodoacetamide for 30 min at 21 °C protected from light. Phosphoric acid was then added to each sample to 1.2%, followed by vortexing. Samples were diluted 6.6-fold in S-TRAP Bind and Wash buffer composed of 90% methanol containing 100 mM tetraethylammonium bromide (TEAB; pH 8) and loaded into an S-TRAP Micro spin column by centrifugation at 4,000*g* at 21 °C until all of the solution passed through the column. Each spin column was subsequently washed with 150 μl of S-TRAP Bind and Wash buffer and centrifuged at 4,000*g* for 30 s at 21 °C. The flow-through was discarded. This wash step was repeated three more times for a total of four washes. Before digestion, spin columns were transferred to clean collection tubes. Porcine trypsin (Promega) in 50 mM TEAB (pH 8) was added to each spin column to a final enzyme:substrate ratio of 1:10, followed by incubation for 2 h at 47 °C without agitation. Digested peptides were eluted from the spin columns by the addition of 40 μl of S-TRAP Elution Buffer A composed of 50 mM TEAB (pH 8) and centrifugation at 4,000*g* for 30 s at 21 °C. A second elution was performed by the addition of equal volumes of S-TRAP Elution Buffer B composed of 0.5% trifluoroacetic acid in water and S-TRAP Elution Buffer C composed of 0.5% trifluoroacetic acid in 50% acetonitrile. The eluates were combined, dried using a speedvac and resuspended in 0.1% formic acid (FA) for subsequent mass spectrometry analysis.

#### Evosep/timsTOF liquid chromatography–tandem mass spectrometry

A high-throughput liquid chromatography–tandem mass spectrometry workflow was implemented as described in Jones et al.^63^. EvoTips (Evosep) were activated by soaking in propanol and washed with 20 μl of 0.1% FA in acetonitrile (solvent B) with centrifugation at 700*g* for 60 s. Tips were conditioned by soaking in propanol until the C18 material appeared pale white, centrifuged at 700*g* for 60 s and equilibrated with 20 μl of 0.1% FA in water (solvent A). To prevent drying out, samples were loaded into tips while equilibrating in solvent A. Peptides were bound to the C18 material by centrifugation at 700*g* for 60 s. Tips were subsequently washed with 20 μl of solvent A and centrifuged at 700*g* for 60 s. Following this, 100 μl of solvent A was added to the tips, which were then centrifuged at 700*g* for 10 s. Liquid chromatography–tandem mass spectrometry analysis was performed immediately. Peptides were analyzed using an Evosep One (Evosep) coupled to a timsTOF Pro mass spectrometer (Bruker), with separation of peptides on a C18 column (150 μm × 80 mm) packed with 1.5-μm beads (PepSep). A gradient length of 21 min at a flow rate of 1 μl min^–1^ was used. The timsTOF Pro mass spectrometer was operated in a parallel accumulation, serial fragmentation (PASEF) mode. Trapped ion mobility spectrometry (TIMS) ion accumulation and ramp times were set to 100 ms, and mass spectra were recorded from *m*/*z* 100 to 1,700. diaPASEF used eight diaPASEF scans per TIMS–mass spectrometry scan, giving a duty cycle of ~1 s. The ion mobility range was set to 0.85–1.3 Vs cm^–2^. Each mass window isolated was *m*/*z* 25 wide, ranging from *m*/*z* 475 to 1,000 and with an ion mobility-dependent collision energy that increased linearly from 27 eV to 45 eV between 0.85 and 1.3 Vs cm^–2^.

#### Data searching

Data-independent acquisition data were searched using default settings in DIA-NN software (version 1.8). Methionine oxidation was set as a variable modification, and cysteine carbamidomethylation was set as a fixed modification. The search allowed for one missed trypsin cleavage site. Samples were searched against the *Homo sapiens* UniProt proteome (retrieved in February 2021) and quantified by label-free quantitation (LFQ).

#### Mass spectrometry data analysis

Raw protein intensities were analyzed in R 4.2.1 (ref. ^64^), with all code documented within R Markdown^65^ at https://github.com/duff-lab-team/AD-EV-characterisation. Reproducibility and package versioning is provided through Singularity/Docker^56^. The following pipeline was used for both protein and peptide LFQ intensities. Intensity data were imported and handled using the Differential Enrichment analysis of Proteomics data package^66^. A total of 6,105 unique proteins were identified across all the fraction groups (Supplementary Fig. 2a). Three fraction 8 samples (A, F and E) were excluded from the analysis because significantly less material was isolated from those donors. After filtering (described in the flowchart below), 6,054 proteins were retained for the imputation pipeline.

Missing data (Supplementary Fig. 2b,c) were handled using a hybrid missing-not-at-random (MNAR) and missing-at-random (MAR) imputation strategy designed to distinguish between proteins that were completely absent from a particular fraction (likely MNAR) and proteins with values missing only from a small number of donors or fractions (likely MAR). This hybrid strategy maximizes the inclusion of differentially expressed proteins (DEPs) across the fractions and avoids exclusion of biologically relevant protein identities (for example, proteins expressed selectively in one or two fraction groups)^67^. This strategy led to a 43% increase in the number of DEPs compared to if the analysis was restricted to proteins without missing values in any of the fractions.

#### Design and implementation of the hybrid imputation strategy

To demonstrate the effectiveness of the hybrid imputation strategy and aid in the selection of MAR versus MNAR missing values, a simulated dataset was produced from a matrix of 6,105 complete case log_2_ (intensity values) based on the mean and standard deviation of proteins in the real dataset^67^. To model the characteristics of the real dataset, 3,000 DEPs and missing values of both MAR and MNAR type were manually assigned to the simulation. The relationship between true missing values across different mean intensities with MAR alone (Supplementary Fig. 3a) versus MNAR + MAR (Supplementary Fig. 3b) was investigated to define a cutoff for MNAR missing values. In the simulation, the MNAR cut-off was set at the bottom of the steep decline in true missing values (log2 intensity value of 12); Supplementary Fig. 3b). This maximized the number of true DEPs captured, the accuracy of their detection and the average adjusted *F*-test *P* value of an analysis of variance across all three fraction groups (Supplementary Fig. 4a). Imputation of the simulated dataset identified 92.97% of the true DEPs with similar accuracy as using the complete cases, while only 69.07% and 24.03% of true DEPs were captured in the unimputed and complete cases (Supplementary Fig. 4b,c). To apply this strategy to the real dataset, MNAR log_2_ (intensity cutoff) values were manually determined for each fraction using the simulation curve as a guide (Supplementary Fig. 3c): fraction 1, 14.9; fraction 2, 14.7; fraction 3, 14.9; fraction 4, 14.3; fraction 5, 14.6; fraction 6, 14.5; fraction 7, 15.3; fraction 8, 15.2. In the real dataset, 827 more proteins were detected as DEPs following imputation (Supplementary Fig. 4d). Data were normalized using a variance stabilizing transformation, and principal component analysis was performed and visualized using the R package ggplot2 (ref. ^68^). The 68% confidence interval was used to draw ellipses surrounding the points for each donor (Fig. 1b).

#### Differential expression and Gene Ontology pathway analysis

Differential expression analysis was performed using moderated *F*- and *t*-statistics from the R package limma^69,70^, with visualization aided by the R packages volcano3D^60^ and ComplexHeatmap^71^. Pairwise contrasts were tested across the three fraction groups using the Benjamini and Hochberg multiple testing correction factor. Proteins were deemed significant at adjusted *P* values of <0.05, without a log fold change cutoff. Gene Ontology analysis (Fig. 1c) was performed using the ssGSEA method from the GSVA R package^72^. After the ssGSEA transformation, the same limma testing procedure was applied to the new matrix, with a more stringent threshold of adjusted *P* < 5 × 10^−4^. To reduce multiple testing burden, data were filtered such that only gene sets with between 5 and 50 genes detected were used. Gene sets with more than 25% overlapping genes and gene sets with directionality modifiers as ‘up/downregulation of’ and so on were discarded. Relevant terms with the highest *F*-test *P* values across the fraction groups were selected for display in Fig. 1c.

### HEK cell model of tau assembly

HEK293T cells (ATCC, CRL-3216) were transduced with lentivirus expressing 1N4R tau with the P301S mutation fused to enhanced YFP at the C terminus. A clonal population was obtained by fluorescence-activated cell sorting and was seeded with PHFs isolated from human AD brain tissue (Extended Data Table 1). The seeded population was again sorted, and clones were selected that constitutively propagated assembled tau, as visualized by the presence of YFP^+^ cytoplasmic puncta. Cells were cultured in complete medium (DMEM; Gibco, 41966029) supplemented with 10% fetal bovine serum (Gibco, 10082-147) and 1% penicillin/streptomycin (Gibco, 15140-122) in flasks coated with 20 μg ml^–1^ poly-d-lysine (Sigma, P6403). Confocal images were acquired using a Leica Stellaris 8 STED microscope with a ×100 oil objective (Extended Data Fig. 4a).

### Isolation of EVs from HEK cell medium

When expanding cells for EV collection, the cells were plated as described above in complete medium and grown to 50% confluency. Serum-containing medium was removed, cells were washed gently in warmed PBS, and the medium was replaced with serum-free Advanced DMEM (Gibco, 12491015) supplemented with 2 mM l-glutamine (Gibco, A2916801) and 1% penicillin/streptomycin (Gibco, 15140-122). The medium was pipetted from cells at 100% confluency and centrifuged for 10 min at 2,000*g* to pellet cells and large debris. Medium supernatants were stored at −80 °C. For each EV isolation, approximately 360 ml of collected medium was centrifuged at 150,000*g* at 4 °C for 16 h to generate a crude EV pellet, which was resuspended in 30% OP and overlayed with 20% and 10% OP layers. Gradients were centrifuged as per the human EVs. All floated material (including the 30–20% interface) was pooled, diluted 1:15 in PBS (Gibco, 10010-015) and centrifuged at 150,000*g* for 3 h to collect an EV pellet.

### NTA particle analysis

At isolation, fresh EVs were partitioned into 3-μl aliquots for particle counting and frozen at −80 °C. Samples were thawed only once before NTA analysis (ZetaView PMX-420 Quatt, Particle Metrix) and immediately diluted in particle-free PBS to the optimal reading range of the instrument (100–150 particles). Samples were diluted in PBS (between 1:100,000 and 1:5,000) and analyzed at 22 °C in scatter mode at the following settings: sensitivity = 80, shutter = 100, frame rate = 30, trace length = 15, bin size = 5 nm and positions per single reading = 11. Code and R Markdown sheets describing the data analysis are deposited at https://github.com/duff-lab-team/AD-EV-characterisation. In brief, data files for each EV sample were aggregated into a single data object and normalized by multiplying the concentration at each size bin by the corresponding dilution factor for that sample. To obtain the number of EVs per milligram of brain tissue (Extended Data Fig. 2e), a scaling factor was applied to normalize each sample to the amount of tissue isolated, and means of three to five technical replicates were calculated. For all other NTA figures, particle counts were used for estimation of the mode, and summing of counts was used to aggregate technical replicates.

### Seeded tau assembly in Tau RD P301S FRET Biosensor cells

Tau RD P301S FRET Biosensor cells (ATCC, CRL-3275) were plated at 30,000 cells per well in 96-well flat-bottomed plates in complete medium (as described above). The cells were allowed to settle undisturbed for 15 min at room temperature before being moved to the incubator and grown at 37 °C with 5% CO_2_. The following day, seed mixes were made in 20 μl per well volumes containing 15 μl of protein dilution (3 μg of EVs in PBS diluted in a final volume of 15 μl of Opti-MEM (31985062, Gibco)) and 5 μl of Lipofectamine dilution (1 μl of Lipofectamine 2000 plus 4 μl of Opti-MEM). Seeding reactions were incubated for 30 min in the cell culture incubator before being added to cells 24 h after plating (cells were ~65–70% confluent at the time of seeding). The seeded cells were maintained undisturbed in the incubator for 48 h before collection for FRET measurements.

At the time of collection, the cell medium was removed from each well using a vacuum manifold for 96-well plates. Trypsin-EDTA (50 μl) was added to each well, and the plate was incubated for 2–3 min at 37 °C. Serum-containing medium (150 μl) was added to each well, and cells were triturated ten times and moved into wells of a fresh v-bottomed 96-well plate. Cells were pelleted at 600*g* for 5 min and resuspended in 150 μl of cold 4% paraformaldehyde in PBS by triturating ten times. Cells were left to fix for 10 min and then repelleted at 600*g* for 5 min. The cells were resuspended in 200 μl of PBS containing 1 mM EDTA. Flow cytometry analysis of FRET signals was performed using a BD LSR Fortessa flow cytometer equipped with a high-throughput sampler using BV510 and BV421 BD filter sets, as previously described^73^. Samples were run on the ‘low’ flow setting (<1,000 events per s), and 50,000 events were captured per well, with three technical replicates for each condition. Integrated FRET densities (percent FRET-positive cells per gated cell population × median fluorescence intensity of the BV510 signal) were obtained using FCS Express 7 Research software (De Novo Software).

### Seeded tau assembly in PS19 mice

All experiments were performed in accordance with protocols approved by the Institutional Animal Care and Use Committee at Columbia University. Three 6-month-old male PS19 mice were injected with pooled AD EVs (fractions 1–8) in the left hemisphere and pooled control EVs (fractions 1–8) in the right hemisphere. Mice were immobilized in a stereotaxic frame (David Kopf Instruments) under continuous isofluorane administration, and stereotaxic injections were made under aseptic conditions using a Hamilton syringe into the hippocampal hilus (anterior–posterior, –2.5 mm; medial–lateral, ±2 mm; dorsal–ventral, −1.8 mm). Each injection contained 10 μg of total EV protein in a volume of 1.5 μl. All animals were carefully monitored and administered analgesics both during and after surgery. Mice were killed 2 months after injection and perfused intracardially with PBS and 10% formalin. Brains were postfixed overnight in 10% formalin and cryoprotected in 30% sucrose for 1 week before TissueTek embedding. Thirty-micron-thick coronal sections spanning the anterior–posterior axis of the hippocampus (bregma –1.5 to 3) were prepared using a Leica cryostat. Free-floating sections were immunolabeled for AT8 using the Mouse-on Mouse (MOM) Elite peroxidase immunodetection kit (Vector Laboratories, PK-2200). Briefly, endogenous peroxidases were quenched in hydrogen peroxide buffer (PBS + 10% methanol + 3% H_2_O_2_) for 15 min at 21 °C. Tissue sections were washed twice in PBS, blocked using the mouse IgG blocking reagent for 1.5 h at 21 °C and washed again three time with PBS. AT8-biotin (Thermo Fisher Scientific, MN1020B) primary antibody was incubated with the sections at a dilution of 1:500 in the MOM diluent overnight at 4 °C. Sections were washed five times in PBS, incubated for 30 min at 21 °C in ABC working solution (Vectastain Elite HRP kit; Vector laboratories, PK-6100) and washed again three times in PBS. 3,3′-Diaminobenzidine (DAB) working solution (DAB Peroxidase Substrate kit; Vector Laboratories, SK-4100) was added to the sections until the color was fully developed (under constant dissection microscope monitoring). Sections were immediately washed twice in PBS to stop the reaction, mounted onto glass coverslips and dehydrated successively in 70% ethanol, 95% ethanol, 100% ethanol and 100% xylene. Coverslips were mounted using Permount (DPX mounting medium) and dried overnight. DAB labeling was assessed every fifth slice (ten slices per animal) at ×20 magnification on a Zeiss Axio Scan microscope. AT8 immunolabeling intensity was quantified from ipsilateral (AD EV-injected) and contralateral (control EV-injected) brain regions (dentate gyrus, CA2/CA3) using ImageJ.

### Isolation of EVs spiked with sarkosyl-insoluble material from AD brain

Sarkosyl-insoluble material enriched with tau PHFs was extracted from 1 g of gray matter from the frontal cortex of an individual who had AD, based on Guo et al.^12^. Gray matter was homogenized in 9 volumes of ice-cold extraction buffer containing 10 mM Tris-HCl (pH 7.4), 0.8 M NaCl, 10% sucrose, 1 mM EDTA, 0.1% sarkosyl and protease/phosphatase inhibitor cocktail (Pierce) using a FastPrep-24 beat-beating homogenizer (MP Biomedicals). The homogenate was centrifuged at 9,296*g* for 10 min at 4 °C using a Ti45 rotor (Beckman). The supernatant was filtered through a 70-μm pore-size cell strainer and retained on ice. The pellet was resuspended in 4.5 volumes of ice-cold extraction buffer and recentrifuged at 9,296*g* for 10 min at 4 °C using a Ti45 rotor (Beckman). The supernatant was filtered through a 70-μm pore-size cell strainer and combined with the first supernatant. The combined filtered supernatant was brought to a final concentration of 1% sarkosyl using 25% sarkosyl in water and incubated for 1 h at 21 °C with stirring at 100 rpm. Following incubation, the supernatant was centrifuged at 139,662*g* for 75 min at 4 °C using a Ti45 rotor (Beckman). The floating lipid layer and supernatant were discarded, and the centrifuge tube was washed twice with 6 ml of PBS, followed by one wash with 2 ml of PBS, taking care not to disturb the pellet. The pellet was transferred to a Ti70 centrifuge tube (Beckman) in 3 ml of PBS and vortexed. The tube was topped up to 23 ml with PBS and centrifuged at 259,939*g* for 30 min at 4 °C using a Ti70 rotor (Beckman). The floating lipid layer and supernatant were discarded, and the pellet was transferred to a 2-ml tube in 50 μl of PBS per g of initial gray matter. The tube was incubated with rocking at 25 rpm for 16 h at 21 °C to soften the pellet before being resuspended by sonication using a Hielscher S26D11X10 Vial-Tweeter-Sonotrode with 0.5-s pulses at 100% amplitude for a total accumulated power of 200 W. The resuspended pellet was transferred to a Ti70 centrifuge tube (Beckman) and centrifuged at 116,982*g* for 40 min at 4 °C using a Ti70 rotor (Beckman). The supernatant was discarded, and the pellet was transferred to a 2-ml tube in 50 μl of PBS per g of initial gray matter and resuspended by sonication using a Hielscher S26D11X10 Vial-Tweeter-Sonotrode in 0.5-s pulses at 100% amplitude for a total accumulated power of 200 W. The resuspended pellet was centrifuged at 10,000*g* for 30 min at 4 °C, and the supernatant, containing extracted tau PHFs, was retained. EVs from 0.3 g of control and AD frontal cortex were isolated as described above but were floated in a simplified OP gradient consisting of 3-ml step gradients of 40, 30, 20, 10 and 0% OP. Immediately before papain digestion, 15 μl of tau PHFs were added to the control EVs to achieve the same amount of tau PHFs that would have been isolated from 0.3 g of AD tissue.

### Tau peptide mapping

Individual tau peptide LFQ intensities were imputed and normalized using the same strategy as the protein dataset and were presented as log_2_ values. Peptides were arranged from N terminus to C terminus, amino acids 1–441. Near-duplicate peptides, corresponding to missed tryptic cleavages and post-translationally modified peptides, were summed to represent the total abundance of sequenced peptides at each amino acid site. Peptides were treated as individual peptides if they were more than 50% unique from a neighboring peptide. A resultant 17 peptides between amino acids 6 and 406 were used in the analysis.

### Sarkosyl extraction of EVs

EV suspensions were pelleted by centrifugation at 100,000*g* for 70 min at 4 °C using a 70.1Ti rotor (Beckman) and resuspended in 20 volumes (wt/vol) of extraction buffer containing 10 mM Tris-HCl (pH 7.5), 0.8 M NaCl, 10% sucrose, 1 mM EGTA and 1% sarkosyl. The resuspended EVs were incubated with rotation for 1 h at 21 °C, followed by centrifugation at 100,000*g* for 70 min at 4 °C. For single-particle cryo-EM, the sarkosyl-insoluble material was further washed by two rounds of resuspension, followed by repelleting by centrifugation at 100,000*g* for 70 min at 4 °C, first using 25 μl of extraction buffer per g of initial tissue used and the same volume of 20 mM Tris-HCl (pH 7.4) and 100 mM NaCl. The final sarkosyl-insoluble pellets were resuspended in 25 μl of 20 mM Tris-HCl (pH 7.4) and 100 mM NaCl per g of initial tissue used. For Fig. 2d, each EV fraction was resuspended in 30 μl of PBS and pooled into three groups: fractions 1–3, 4–6 and 7 and 8. Equal volumes of each fraction group were sarkosyl extracted as described above but underwent a 10-min clearing spin at 10,000*g* after the sarkosyl incubation. An additional wash in 1% sarkosyl buffer was also performed to clear the pellets of lipids. Final sarkosyl-insoluble pellets were sonicated at 65% amplitude for 2 min in 1× Laemmli sample buffer (Bio-Rad, 1610747) containing 50 mM DTT and heated at 95 °C for 5 min before electrophoresis.

### Proteinase K protection assay

Directly after centrifugation, each freshly prepared EV pellet was resuspended in 30 μl of PBS. Fractions 1–3, 4–6 and 7 and 8 were pooled and brought to 1 μg µl^–1^ total protein in PBS. Protease reactions were set up in a total volume of 15 μl with 1.5 μl of the pooled EV sample (1.5 μg of total protein) and 1 μl of 15 ng μl^–1^ proteinase K stock (Thermo Fisher Scientific, EO0491) to maintain a ratio of 10 ng of proteinase K:1 μg of total protein. For EV permeabilization, 1 μl of 1% Triton X-100 stock was added. Reactions were incubated at 37 °C for 30 min and stopped by the addition of 15 μl of 2× Laemmli sample buffer + 2× HALT protease and phosphatase solution (Thermo Fisher Scientific, 78440) and heating for 5 min at 95 °C. The reactions were resolved by SDS–PAGE and immunoblotted with antibody TauC at a dilution of 1:5,000.

### EV fractionation and carbonate membrane purification

EVs were isolated as described above, however, before flotation in the OP gradient, the crude EV pellet was resuspended in 1 ml of PBS containing 0.1 M DTT and incubated at 21 °C for 30 min with end-over-end rotation. DTT-treated EVs were pelleted at 100,000*g* for 70 min at 4 °C in a TLA100 rotor (Beckman). The supernatant, containing DTT-labile EV surface proteins, was concentrated to under 100 μl in 0.5-ml, 3-kDa molecular weight cutoff centrifugal concentrators (Millipore, UFC500396; surface sample in Fig. 4c). The pelleted DTT-treated EVs were resuspended in 5 ml of 40% OP, layered beneath a step gradient of 2.5 ml each of 20, 10 and 0% OP in Buffer B containing 0.1 M DTT and centrifuged at 200,000*g* for 16 h at 4 °C. The top 11 ml and the bottom 2 ml of the gradient were transferred to separate 60-ml 45Ti ultracentrifuge tubes (Beckman), topped up with PBS and centrifuged at 100,000*g* for 70 min at 4 °C. The pellet from the 2-ml fraction from the bottom of the gradient was resuspended in 100 μl of TBS (pellet sample in Fig. 4c). The pellet from the 11-ml fraction from the top of the gradient was resuspended in 1.1 ml of hypotonic buffer consisting of 5 mM potassium phosphate, passed through a 26-gauge needle 20 times to lyse EVs and extract luminal proteins and centrifuged at 150,000*g* for 3 h at 4 °C in a TLA100 rotor. The supernatant, containing luminal proteins, was concentrated to under 100 μl in 0.5-ml 3-kDa molecular weight cutoff centrifugal concentrators (luminal sample in Fig. 4c). The pellet, containing EV membranes, was resuspended in 5 ml of 40% OP and layered beneath another step gradient as described before but in the absence of DTT. The gradient was centrifuged and fractionated as described before, except that the pellet collected from the 11-ml fraction from the top of the gradient was resuspended in 1.1 ml of 0.1 M sodium carbonate buffer (pH 11) and incubated for 30 min at 21 °C to extract all but integral membrane proteins from the EV membranes. The carbonate-stripped membranes were collected by centrifugation at 150,000*g* for 3 h at 4 °C in a TLA100 rotor. The pellet was subjected to a final OP step gradient without DTT, and the 11-ml fraction from the top of the gradient was pelleted at 150,000*g* for 3 h. The final pellet was resuspended in 100 μl of TBS with protease inhibitors (membrane sample in Fig. 4c). Equal proportions of each compartment subfraction were resolved by SDS–PAGE and immunoblotted with antibody TauC at a dilution of 1:5,000.

### Cryo-EM assessment of EVs associated with tau filaments

EVs associated and not associated with tau filaments were manually identified in medium-magnification maps of entire EM grids using cryo-EM. EVs associated with tau filaments were verified using cryo-ET. To estimate the proportion of EVs associated with tau filaments in pooled EV fractions 4–6, average counts from two grid squares from two different EM grids were taken.

### Cryo-ET

Pooled EV fractions 4–6 isolated from 0.8 g of tissue were diluted 1:5 in PBS containing a 1:3 dilution of 10-nm gold-conjugated BSA (BBI solutions), applied to glow-discharged 2/2-μm holey carbon-coated 200-mesh gold grids (Quantifoil) and plunge-frozen in liquid ethane using a Vitrobot Mark IV (Thermo Fisher Scientific). Tilt series were acquired using a 300-keV Titan Krios microscope (Thermo Fisher Scientific) equipped with a K3 detector (Gatan) and a GIF-quantum energy filter (Gatan) operated with a slit width of 20 eV. Tilt series were acquired at a pixel size of 2.13 Å or 1.89 Å at a target defocus of −3 μm to −6 μm. A dose-symmetric acquisition scheme was implemented in Serial EM over a tilt range of ±60° at 3° increments^74,75^. Each tilt received a dose of 3.17 e^−^ Å^–2^ fractionated over eight movie frames, resulting in a total dose of 130 e^−^ Å^–2^ per tilt series.

### Tomogram processing and analyses

Gain correction, motion correction and contrast transfer function (CTF) estimation were performed in Warp^76^. Tilt series alignment using the 10-nm gold-conjugated BSA as fiducials was performed in IMOD^77^. Tomograms were reconstructed in Fourier space at a pixel size of 8 Å or 12 Å, filtered using Wiener-like deconvolution and denoised using the Noise2Noise machine learning principle^78^, all in Warp. For filament and EV measurements, IMOD was used to perform gain correction, motion correction, contrast function estimation and tomogram reconstruction using weighted backprojection at a pixel size of 8.51 Å or 7.46 Å. Filament widths and helical crossover distances were measured manually. Three-dimensional representation and segmentation were performed using napari-tomoslice (https://github.com/alisterburt/napari-tomoslice) in Napari^79^.

### Subtomogram averaging

Subtomogram averaging was performed based on Burt et al.^80^, with adaptations for helical reconstruction. Tomograms were reconstructed in Warp at a pixel size of 16 Å to aid in the visualization of tau filaments. Initial processing steps were performed in Dynamo^81^. Filaments were manually picked using the filament with torsion model, and 17,323 subtomograms were extracted using a box size of 64 pixels at an intersubtomogram distance of 16 Å. An initial reference was created by aligning and averaging a random subset of 250 subtomograms and was subsequently used to align and average all subtomograms. Subtomograms within 16 Å of one another were reduced to a single subtomogram using the remove_duplicates.m script (https://github.com/teamtomo/teamtomo.github.io/tree/master/walkthroughs/EMPIAR-10164/scripts). The metadata table for the remaining 6,133 subtomograms was converted into the Self-defining Text Archiving and Retrieval (STAR) format using the dynamo2m package (https://github.com/alisterburt/dynamo2m). The filament number (Helical Tube ID) for each subtomogram, specified in column 21 of the Dynamo metadata table, was added to the STAR file using the Starparser package (https://github.com/sami-chaaban/starparser). The subtomograms were then re-extracted in Warp at a pixel size of 6 Å and a box size of 204 pixels. Subsequent processing was performed using helical reconstruction methods in RELION-3.1 (ref. ^82^). An initial model was generated by averaging a random subset of 500 subtomograms using relion_reconstruct. Three-dimensional autorefinement followed by 3D classification were then performed without masking or applying symmetry. This yielded reconstructions corresponding to filament types resembling PHFs and SFs. The helical crossover distances were measured from the reconstructions and used to calculate the helical twists for a helical rise of 4.7 Å. Helical symmetry was then applied in all subsequent steps. Three-dimensional classification was repeated to obtain final subsets of subtomograms for PHF and SF types, followed by unmasked and masked 3D autorefinements. The subtomograms were then re-extracted in Warp at pixel sizes of 3.1 Å (PHFs) and 3 Å (SFs), with respective box sizes of 165 pixels and 170 pixels. Three-dimensional autorefinement was repeated, with C2 symmetry imposed for the PHF type. The final reconstructions were sharpened using the standard postprocessing procedures in RELION-3.1, and overall resolutions were estimated at a Fourier shell correlation of 0.143 between the two independently refined half-maps using phase randomization to correct for convolution effects of a generous, soft-edged solvent mask. Helical symmetry was imposed using the RELION Helix Toolbox. Rigid-body fitting of models of PHFs from EVs and SFs from primary age-related tauopathy (Protein Data Bank (PDB) ID 7NRS) was performed in ChimeraX^83^.

### Single-particle cryo-EM

Sarkosyl-insoluble extracts were centrifuged at 10,000*g* for 10 min at 4 °C. The supernatants were retained and centrifuged at 100,000*g* for 1 h at 4 °C. The pellets were resuspended in 30 μl of 20 mM Tris-HCl (pH 7.4) containing 100 mM NaCl and centrifuged at 3,000*g* for 30 s at 21 °C. The supernatants were retained and applied to glow-discharged 1.2/1.3-μm holey carbon-coated 300-mesh gold grids (Quantifoil) and plunge-frozen in liquid ethane using a Vitrobot Mark IV (Thermo Fisher Scientific). Images for the first EV dataset were acquired using a 300-keV Titan Krios microscope (Thermo Fisher Scientific) equipped with a K2 detector (Gatan) at the European Synchrotron Radiation Facility (ESRF)^84^. Images for the second EV dataset and the cell dataset were acquired using a 300-keV Titan Krios microscope (Thermo Fisher Scientific) equipped with a K3 detector (Gatan) at the MRC Laboratory of Molecular Biology. GIF-quantum energy filters (Gatan) operated at slit widths of 20 eV and aberration-free image shift within the EPU software (Thermo Fisher Scientific) were used during image acquisition. Further details are provided in Extended Data Table 2.

### Helical reconstruction

Movie frames were gain corrected, aligned, dose weighted and summed using the motion correction program in RELION-4.0 (ref. ^85^). The motion-corrected micrographs were used to estimate the CTF using CTFFIND-4.1 (ref. ^86^). All subsequent image processing was performed using helical reconstruction methods in RELION-4.0 (ref. ^87^). The filaments were picked manually, and reference-free 2D classification was performed to remove suboptimal segments. Initial 3D reference models were generated de novo by producing sinograms from 2D class averages as previously described^88^. Three-dimensional autorefinements with optimization of the helical twist were performed, followed by two cycles of Bayesian polishing and CTF refinement^85^. Three-dimensional classification was used to further remove suboptimal segments, after which the 3D autorefinement, Bayesian polishing and CTF refinement procedures were repeated. The final reconstructions were sharpened using the standard postprocessing procedures in RELION-4.0, and overall resolutions were estimated at a Fourier shell correlation of 0.143 between the two independently refined half-maps using phase randomization to correct for convolution effects of a generous, soft-edged solvent mask^89^. Local resolution estimates were obtained using the same phase randomization procedure but with a soft spherical mask that was moved over the entire map. Helical symmetry was imposed using the RELION Helix Toolbox. Further details are provided in Extended Data Table 2.

### Atomic model building and refinement

The published atomic model of PHFs from the frontotemporal cortex of an individual with sporadic AD (PDB: 6HRE) was fitted to the final reconstructions using ChimeraX. The fitted models were subsequently refined in real-space using COOT^90^. Rebuilding using molecular dynamics was performed in ISOLDE^91^. The models were refined in Fourier space using REFMAC5 (ref. ^92^), with appropriate symmetry constraints defined using Servalcat^93^. To confirm the absence of overfitting, the model was shaken, refined in Fourier space against the first half-map using REFMAC5 and compared to the second half-map (Extended Data Fig. 8f). Geometry was validated using MolProbity^94^. Molecular graphics and analyses were performed in ChimeraX. Model statistics are provided in Extended Data Table 2.

### Reporting summary

Further information on research design is available in the Nature Portfolio Reporting Summary linked to this article.

## Online content

Any methods, additional references, Nature Portfolio reporting summaries, source data, extended data, supplementary information, acknowledgements, peer review information; details of author contributions and competing interests; and statements of data and code availability are available at 10.1038/s41593-024-01801-5.

## Supplementary information

Supplementary Information Supplementary Figs. 1–4.

Reporting Summary

Supplementary Data 1 This Excel spreadsheet contains the protein lists used to generate Figs. 1 and 2b and Extended Data Figs. 1 and 3a–c. Raw and processed mass spectrometry data can be found at https://github.com/duff-lab-team/AD-EV-characterisation.

## Source data

Source Data Unprocessed western blots for Figs. 1, 2c,d, 3c and 4c and Extended Data Figs. 4c and 5.

## Figures and Tables

**Extended Data Table 1:**
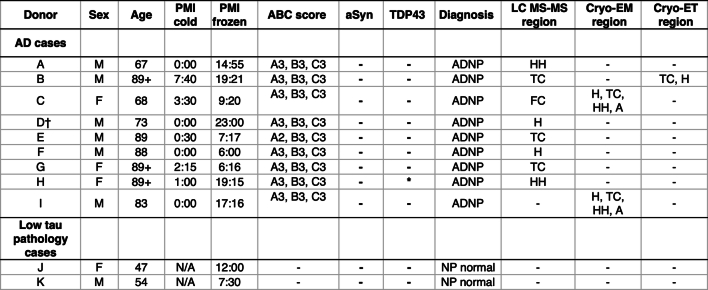
Human Brain Tissue Samples

PMI cold: postmortem interval – time to cold (h:min); PMI frozen: postmortem interval – time to frozen (h:min). All AD cases were diagnosed on autopsy as ADNC (Alzheimer’s Disease Neuropathologic Changes), were classified as Braak stage VI, with high likelihood that dementia was due to AD. All AD cases were categorized as A3 (Thal phase for Aβ plaques 4 or 5), B3 (Braak stage for tau neurofibrillary tangles V or VI), C3 (CERAD neuritic plaque score of ‘Frequent’) except case E (A2). aMI: acute myocardial infarction; TC: temporal cortex; HH: head of hippocampus; H: hippocampus, FC: frontal cortex; A: amygdala; PVC: primary visual cortex; Cryo-EM: Single-particle cryo-electron microscopy; Cryo-ET; cryo-electron tomography; aSyn: concomitant alpha-synuclein pathology; TDP43: concomitant TDP-43 pathology; NP normal: neuropathologically normal. *Rare neurons in temporal regions with ubiquitinated TDP-43 labelling. †AD case used to generate seeded HEK cell model of EV release (region FC). All human AD specimens were fresh frozen and sourced from the New York Brain Bank (NYBB) at Columbia University (Alzheimer’s Disease Research Center). Low tau pathology cases were sourced from the University of Miami Brain Endowment Bank.

**Extended Data Table 2:**
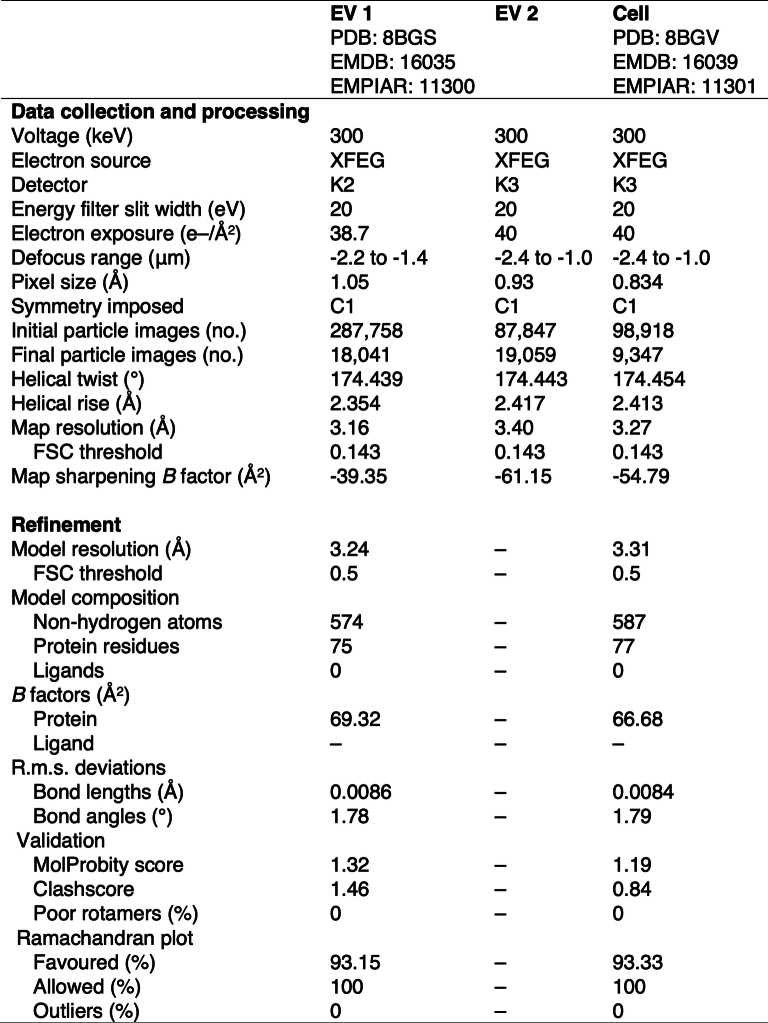
Cryo-EM data collection, refinement and validation statistics

Cryo-EM data collection, refinement and validation statistics.

## Data Availability

The mass spectrometry proteomics data have been deposited to the ProteomeXchange Consortium via the PRIDE^95^ partner repository with the dataset identifier PXD037708. Data analysis for mass spectrometry proteomics and NTA datasets and supplemental figures describing the imputation strategy are accessible at https://github.com/duff-lab-team/AD-EV-characterisation. The *H. sapiens* proteome is available from UniProt under accession code UP000005640. The tomogram shown in Fig. 3a has been deposited to the Electron Microscopy Data Bank under the accession number EMD-16064. Single-particle cryo-EM datasets have been deposited to the Electron Microscopy Public Image Archive under the following accession numbers: 11300 for the EV dataset and 11301 for the cellular dataset. Single-particle cryo-EM maps have been deposited to the Electron Microscopy Data Bank under the following accession numbers: EMD-16035 for the EV map and EMD-16039 for the cellular map. The atomic models have been deposited to the PDB under the following accession numbers: 8BGS for the EV model and 8BGV for the cellular model. Any other data are available from the corresponding authors upon request. Source data are provided with this paper.

## Code availability

All R Markdown code required to reproduce the mass spectrometry analyses for Fig. 1b,c and Extended Data Figs. 1, 2e–h, 3 and 4b are accessible without restriction at https://github.com/duff-lab-team/AD-EV-characterisation.

## References

[1] Fitzpatrick, A. W. P. et al. Cryo-EM structures of tau filaments from Alzheimer’s disease. *Nature* **547**, 185–190 (2017). 28678775 10.1038/nature23002 PMC5552202

[2] Falcon, B. et al. Tau filaments from multiple cases of sporadic and inherited Alzheimer’s disease adopt a common fold. *Acta Neuropathol.* **136**, 699–708 (2018). 30276465 10.1007/s00401-018-1914-z PMC6208733

[3] La Joie, R. et al. Prospective longitudinal atrophy in Alzheimer’s disease correlates with the intensity and topography of baseline tau-PET. *Sci. Transl. Med.* **12**, eaau5732 (2020). 31894103 10.1126/scitranslmed.aau5732 PMC7035952

[4] Nelson, P. T. et al. Correlation of Alzheimer disease neuropathologic changes with cognitive status: a review of the literature. *J. Neuropathol. Exp. Neurol.* **71**, 362–381 (2012). 22487856 10.1097/NEN.0b013e31825018f7 PMC3560290

[5] Lee, V. M.-Y., Goedert, M. & Trojanowski, J. Q. Neurodegenerative tauopathies. *Annu. Rev. Neurosci.* **24**, 1121–1159 (2001). 11520930 10.1146/annurev.neuro.24.1.1121

[6] Scheres, S. H. W., Ryskeldi-Falcon, B. & Goedert, M. Molecular pathology of neurodegenerative diseases by cryo-EM of amyloids. *Nature* **621**, 701–710 (2023). 37758888 10.1038/s41586-023-06437-2

[7] Spillantini, M. G. et al. Mutation in the tau gene in familial multiple system tauopathy with presenile dementia. *Proc. Natl Acad. Sci. USA* **95**, 7737–7741 (1998). 9636220 10.1073/pnas.95.13.7737 PMC22742

[8] Hutton, M. et al. Association of missense and 5′-splice-site mutations in tau with the inherited dementia FTDP-17. *Nature* **393**, 702–705 (1998). 9641683 10.1038/31508

[9] Poorkaj, P. et al. Tau is a candidate gene for chromosome 17 frontotemporal dementia. *Ann. Neurol.* **43**, 815–825 (1998). 9629852 10.1002/ana.410430617

[10] Pascoal, T. A. et al. Longitudinal 18F-MK-6240 tau tangles accumulation follows Braak stages. *Brain* **144**, 3517–3528 (2021). 34515754 10.1093/brain/awab248 PMC8677534

[11] Braak, H. & Braak, E. Neuropathological stageing of Alzheimer-related changes. *Acta Neuropathol.* **82**, 239–259 (1991). 1759558 10.1007/BF00308809

[12] Guo, J. L. et al. Unique pathological tau conformers from Alzheimer’s brains transmit tau pathology in nontransgenic mice. *J. Exp. Med.* **213**, 2635–2654 (2016). 27810929 10.1084/jem.20160833 PMC5110027

[13] Frost, B., Jacks, R. L. & Diamond, M. I. Propagation of tau misfolding from the outside to the inside of a cell. *J. Biol. Chem.* **284**, 12845–12852 (2009). 19282288 10.1074/jbc.M808759200 PMC2676015

[14] Clavaguera, F. et al. Transmission and spreading of tauopathy in transgenic mouse brain. *Nat. Cell Biol.* **11**, 909–913 (2009). 19503072 10.1038/ncb1901 PMC2726961

[15] Wu, J. W. et al. Neuronal activity enhances tau propagation and tau pathology in vivo. *Nat. Neurosci.* **19**, 1085–1092 (2016). 27322420 10.1038/nn.4328 PMC4961585

[16] Budnik, V., Ruiz-Cañada, C. & Wendler, F. Extracellular vesicles round off communication in the nervous system. *Nat. Rev. Neurosci.* **17**, 160–172 (2016). 26891626 10.1038/nrn.2015.29 PMC4989863

[17] van Niel, G. et al. Challenges and directions in studying cell–cell communication by extracellular vesicles. *Nat. Rev. Mol. Cell Biol.* **23**, 369–382 (2022). 35260831 10.1038/s41580-022-00460-3

[18] Korkut, C. et al. Regulation of postsynaptic retrograde signaling by presynaptic exosome release. *Neuron* **77**, 1039–1046 (2013). 23522040 10.1016/j.neuron.2013.01.013 PMC3626103

[19] Fauré, J. et al. Exosomes are released by cultured cortical neurones. *Mol. Cell. Neurosci.* **31**, 642–648 (2006). 16446100 10.1016/j.mcn.2005.12.003

[20] Tian, T. et al. Exosome uptake through clathrin-mediated endocytosis and macropinocytosis and mediating miR-21 delivery. *J. Biol. Chem.* **289**, 22258–22267 (2014). 24951588 10.1074/jbc.M114.588046 PMC4139237

[21] Joshi, B. S., de Beer, M. A., Giepmans, B. N. G. & Zuhorn, I. S. Endocytosis of extracellular vesicles and release of their cargo from endosomes. *ACS Nano* **14**, 4444–4455 (2020). 32282185 10.1021/acsnano.9b10033 PMC7199215

[22] Miyoshi, E. et al. Exosomal tau with seeding activity is released from Alzheimer’s disease synapses, and seeding potential is associated with amyloid β. *Lab. Invest.* **101**, 1605–1617 (2021). 34462532 10.1038/s41374-021-00644-z PMC8590975

[23] Saman, S. et al. Exosome-associated tau is secreted in tauopathy models and is selectively phosphorylated in cerebrospinal fluid in early Alzheimer disease. *J. Biol. Chem.* **287**, 3842–3849 (2012). 22057275 10.1074/jbc.M111.277061 PMC3281682

[24] Crotti, A. et al. BIN1 favors the spreading of tau via extracellular vesicles. *Sci. Rep.* **9**, 9477 (2019). 31263146 10.1038/s41598-019-45676-0 PMC6603165

[25] Ruan, Z. et al. Alzheimer’s disease brain-derived extracellular vesicles spread tau pathology in interneurons. *Brain* **144**, 288–309 (2021). 33246331 10.1093/brain/awaa376 PMC7880668

[26] Leroux, E. et al. Extracellular vesicles: major actors of heterogeneity in tau spreading among human tauopathies. *Mol. Ther.* **30**, 782–797 (2021). 34563677 10.1016/j.ymthe.2021.09.020 PMC8821971

[27] Polanco, J. C., Hand, G. R., Briner, A., Li, C. & Götz, J. Exosomes induce endolysosomal permeabilization as a gateway by which exosomal tau seeds escape into the cytosol. *Acta Neuropathol.* **141**, 235–256 (2021). 33417012 10.1007/s00401-020-02254-3 PMC7847444

[28] Wang, Y. et al. The release and *trans*-synaptic transmission of tau via exosomes. *Mol. Neurodegener.* **12**, 5 (2017). 28086931 10.1186/s13024-016-0143-y PMC5237256

[29] Asai, H. et al. Depletion of microglia and inhibition of exosome synthesis halt tau propagation. *Nat. Neurosci.* **18**, 1584–1593 (2015). 26436904 10.1038/nn.4132 PMC4694577

[30] Théry, C. et al. Minimal information for studies of extracellular vesicles 2018 (MISEV2018): a position statement of the International Society for Extracellular Vesicles and update of the MISEV2014 guidelines. *J. Extracell. Vesicles* **7**, 1535750 (2018). 30637094 10.1080/20013078.2018.1535750 PMC6322352

[31] Welsh, J. A. et al. Minimal information for studies of extracellular vesicles (MISEV2023): from basic to advanced approaches. *J. Extracell. Vesicle* **13**, e12404 (2024). 10.1002/jev2.12404 PMC10850029 38326288

[32] Holmes, B. B. et al. Proteopathic tau seeding predicts tauopathy in vivo. *Proc. Natl Acad. Sci. USA* **111**, E4376–E4385 (2014). 25261551 10.1073/pnas.1411649111 PMC4205609

[33] Yoshiyama, Y. et al. Synapse loss and microglial activation precede tangles in a P301S tauopathy mouse model. *Neuron* **53**, 337–351 (2007). 17270732 10.1016/j.neuron.2007.01.010

[34] Carnell-Morris, P., Tannetta, D., Siupa, A., Hole, P. & Dragovic, R. in *Extracellular Vesicles*, Vol. 1660 (eds Kuo, W. P. & Jia, S.) 153–173 (Springer, 2017). 10.1007/978-1-4939-7253-1_13 28828655

[35] Zabeo, D. et al. Exosomes purified from a single cell type have diverse morphology. *J. Extracell. Vesicles* **6**, 1329476 (2017). 28717422 10.1080/20013078.2017.1329476 PMC5505001

[36] D’Acunzo, P. et al. Mitovesicles are a novel population of extracellular vesicles of mitochondrial origin altered in Down syndrome. *Sci. Adv.* **7**, eabe5085 (2021). 33579698 10.1126/sciadv.abe5085 PMC7880603

[37] Johnson, E. C. B. et al. Large-scale deep multi-layer analysis of Alzheimer’s disease brain reveals strong proteomic disease-related changes not observed at the RNA level. *Nat. Neurosci.* **25**, 213–225 (2022). 35115731 10.1038/s41593-021-00999-y PMC8825285

[38] Jicha, G. A., Bowser, R., Kazam, I. G. & Davies, P. Alz-50 and MC-1, a new monoclonal antibody raised to paired helical filaments, recognize conformational epitopes on recombinant tau. *J. Neurosci. Res.* **48**, 128–132 (1997). 9130141 10.1002/(sici)1097-4547(19970415)48:2<128::aid-jnr5>3.0.co;2-e

[39] Yagishita, S., Itoh, Y., Nan, W. & Amano, N. Reappraisal of the fine structure of Alzheimer’s neurofibrillary tangles. *Acta Neuropathol.* **54**, 239–246 (1981). 7257733 10.1007/BF00687747

[40] Jeppesen, D. K. et al. Reassessment of exosome composition. *Cell* **177**, 428–445 (2019). 30951670 10.1016/j.cell.2019.02.029 PMC6664447

[41] Valcz, G. et al. En bloc release of MVB-like small extracellular vesicle clusters by colorectal carcinoma cells. *J. Extracell. Vesicles* **8**, 1596668 (2019). 31007874 10.1080/20013078.2019.1596668 PMC6461071

[42] Petersen, J. D., Mekhedov, E., Kaur, S., Roberts, D. D. & Zimmerberg, J. Endothelial cells release microvesicles that harbour multivesicular bodies and secrete exosomes. *J. Extracell. Biol.* **2**, e79 (2023). 38939691 10.1002/jex2.79 PMC11080864

[43] Kim, H., Botelho, S. C., Park, K. & Kim, H. Use of carbonate extraction in analyzing moderately hydrophobic transmembrane proteins in the mitochondrial inner membrane. *Protein Sci.* **24**, 2063–2069 (2015). 26435163 10.1002/pro.2817 PMC4815233

[44] Lövestam, S. et al. Disease-specific tau filaments assemble via polymorphic intermediates. *Nature* **625**, 119–125 (2024). 38030728 10.1038/s41586-023-06788-w PMC10764278

[45] Hessvik, N. P. et al. PIKfyve inhibition increases exosome release and induces secretory autophagy. *Cell. Mol. Life Sci.* **73**, 4717–4737 (2016). 27438886 10.1007/s00018-016-2309-8 PMC11108566

[46] Villarroya-Beltri, C. et al. ISGylation controls exosome secretion by promoting lysosomal degradation of MVB proteins. *Nat. Commun.* **7**, 13588 (2016). 27882925 10.1038/ncomms13588 PMC5123068

[47] Debnath, J. & Leidal, A. M. Secretory autophagy during lysosome inhibition (SALI). *Autophagy* **18**, 2498–2499 (2022). 10.1080/15548627.2022.2095788 PMC9542525 35786367

[48] Abdulrahman, B. A., Abdelaziz, D. H. & Schatzl, H. M. Autophagy regulates exosomal release of prions in neuronal cells. *J. Biol. Chem.* **293**, 8956–8968 (2018). 29700113 10.1074/jbc.RA117.000713 PMC5995502

[49] Fussi, N. et al. Exosomal secretion of α-synuclein as protective mechanism after upstream blockage of macroautophagy. *Cell Death Dis.* **9**, 757 (2018). 29988147 10.1038/s41419-018-0816-2 PMC6037700

[50] Zhang, W. et al. Impairment of the autophagy–lysosomal pathway in Alzheimer’s diseases: pathogenic mechanisms and therapeutic potential. *Acta Pharm. Sin. B* **12**, 1019–1040 (2022). 35530153 10.1016/j.apsb.2022.01.008 PMC9069408

[51] Clayton, K. et al. Plaque associated microglia hyper-secrete extracellular vesicles and accelerate tau propagation in a humanized APP mouse model. *Mol. Neurodegener.* **16**, 18 (2021). 33752701 10.1186/s13024-021-00440-9 PMC7986521

[52] Otero-Garcia, M. et al. Molecular signatures underlying neurofibrillary tangle susceptibility in Alzheimer’s disease. *Neuron* **110**, 2929–2948 (2022). 35882228 10.1016/j.neuron.2022.06.021 PMC9509477

[53] Falcon, B. et al. Structures of filaments from Pick’s disease reveal a novel tau protein fold. *Nature* **561**, 137–140 (2018). 30158706 10.1038/s41586-018-0454-y PMC6204212

[54] Wischik, C. M. et al. Isolation of a fragment of tau derived from the core of the paired helical filament of Alzheimer disease. *Proc. Natl Acad. Sci. USA* **85**, 4506–4510 (1988). 3132715 10.1073/pnas.85.12.4506 PMC280459

[55] Wang, Y. P., Biernat, J., Pickhardt, M., Mandelkow, E. & Mandelkow, E.-M. Stepwise proteolysis liberates tau fragments that nucleate the Alzheimer-like aggregation of full-length tau in a neuronal cell model. *Proc. Natl Acad. Sci. USA* **104**, 10252–10257 (2007). 17535890 10.1073/pnas.0703676104 PMC1891218

[56] Takeda, S. et al. Neuronal uptake and propagation of a rare phosphorylated high-molecular-weight tau derived from Alzheimer’s disease brain. *Nat. Commun.* **6**, 8490 (2015). 26458742 10.1038/ncomms9490 PMC4608380

[57] Jackson, S. J. et al. Short fibrils constitute the major species of seed-competent tau in the brains of mice transgenic for human P301S tau. *J. Neurosci.* **36**, 762–772 (2016). 26791207 10.1523/JNEUROSCI.3542-15.2016 PMC4719013

[58] Martinez, P. et al. Bassoon contributes to tau-seed propagation and neurotoxicity. *Nat. Neurosci.* **25**, 1597–1607 (2022). 36344699 10.1038/s41593-022-01191-6 PMC9708566

[59] Jones, E. M. et al. Interaction of tau protein with model lipid membranes induces tau structural compaction and membrane disruption. *Biochemistry* **51**, 2539–2550 (2012). 22401494 10.1021/bi201857v PMC3319454

[60] Vonsattel, J. P. G., Del Amaya, M. P. & Keller, C. E. Twenty-first century brain banking. Processing brains for research: the Columbia University methods. *Acta Neuropathol.* **115**, 509–532 (2008). 17985145 10.1007/s00401-007-0311-9 PMC2292479

[61] D’Acunzo, P. et al. Isolation of mitochondria-derived mitovesicles and subpopulations of microvesicles and exosomes from brain tissues. *Nat. Protoc.* **17**, 2517–2549 (2022). 35962195 10.1038/s41596-022-00719-1 PMC9633367

[62] Crescitelli, R., Lässer, C. & Lötvall, J. Isolation and characterization of extracellular vesicle subpopulations from tissues. *Nat. Protoc.* **16**, 1548–1580 (2021). 33495626 10.1038/s41596-020-00466-1

[63] Jones, H. B. L. et al. ABPP-HT*—deep meets fast for activity-based profiling of deubiquitylating enzymes using advanced DIA mass spectrometry methods. *Int. J. Mol. Sci.* **23**, 3263 (2022). 35328685 10.3390/ijms23063263 PMC8955990

[64] R Core Team. *R: A Language and Environment for Statistical Computer* (R Foundation for Statistical Computing, 2022).

[65] Allaire, J. rmarkdown: dynamic documents for R. *GitHub* https://github.com/rstudio/rmarkdown (2021).

[66] Zhang, X. et al. Proteome-wide identification of ubiquitin interactions using UbIA-MS. *Nat. Protoc.* **13**, 530–550 (2018). 29446774 10.1038/nprot.2017.147

[67] Gardner, M. L. & Freitas, M. A. Multiple imputation approaches applied to the missing value problem in bottom-up proteomics. *Int. J. Mol. Sci.* **22**, 9650 (2021). 34502557 10.3390/ijms22179650 PMC8431783

[68] Wickham, H. *Ggplot2: Elegant Graphics for Data Analysis* (Springer International Publishing, 2016).

[69] Ritchie, M. E. et al. limma powers differential expression analyses for RNA-sequencing and microarray studies. *Nucleic Acids Res.* **43**, e47 (2015). 25605792 10.1093/nar/gkv007 PMC4402510

[70] Phipson, B., Lee, S., Majewski, I. J., Alexander, W. S. & Smyth, G. K. Robust hyperparameter estimation protects against hypervariable genes and improves power to detect differential expression. *Ann. Appl. Stat.* **10**, 946–963 (2016). 28367255 10.1214/16-AOAS920 PMC5373812

[71] Gu, Z., Eils, R. & Schlesner, M. Complex heatmaps reveal patterns and correlations in multidimensional genomic data. *Bioinformatics* **32**, 2847–2849 (2016). 27207943 10.1093/bioinformatics/btw313

[72] Hänzelmann, S., Castelo, R. & Guinney, J. GSVA: gene set variation analysis for microarray and RNA-seq data. *BMC Bioinformatics* **14**, 7 (2013). 23323831 10.1186/1471-2105-14-7 PMC3618321

[73] Furman, J. L., Holmes, B. B. & Diamond, M. I. Sensitive detection of proteopathic seeding activity with FRET flow cytometry. *J. Vis. Exp.* **8**, e53205 (2015). 10.3791/53205 PMC4692784 26710240

[74] Mastronarde, D. N. Automated electron microscope tomography using robust prediction of specimen movements. *J. Struct. Biol.* **152**, 36–51 (2005). 16182563 10.1016/j.jsb.2005.07.007

[75] Hagen, W. J. H., Wan, W. & Briggs, J. A. G. Implementation of a cryo-electron tomography tilt-scheme optimized for high resolution subtomogram averaging. *J. Struct. Biol.* **197**, 191–198 (2017). 27313000 10.1016/j.jsb.2016.06.007 PMC5287356

[76] Tegunov, D. & Cramer, P. Real-time cryo-electron microscopy data preprocessing with Warp. *Nat. Methods* **16**, 1146–1152 (2019). 31591575 10.1038/s41592-019-0580-y PMC6858868

[77] Kremer, J. R., Mastronarde, D. N. & McIntosh, J. R. Computer visualization of three-dimensional image data using IMOD. *J. Struct. Biol.* **116**, 71–76 (1996). 8742726 10.1006/jsbi.1996.0013

[78] Lehtinen, J. et al. Noise2Noise: learning image restoration without clean data. In *Proc. 35th International Conference on Machine Learning* (eds. Dy, J. & Krause, A.) 2965–2974 (International Conference on Machine Learning, 2018).

[79] Sofroniew, N. et al. napari: a multi-dimensional image viewer for Python. https://napari.org/0.4.15/index.html (2022).

[80] Burt, A., Gaifas, L., Dendooven, T. & Gutsche, I. A flexible framework for multi-particle refinement in cryo-electron tomography. *PLoS Biol.* **19**, e3001319 (2021). 34437530 10.1371/journal.pbio.3001319 PMC8389456

[81] Castaño-Díez, D., Kudryashev, M., Arheit, M. & Stahlberg, H. Dynamo: a flexible, user-friendly development tool for subtomogram averaging of cryo-EM data in high-performance computing environments. *J. Struct. Biol.* **178**, 139–151 (2012). 22245546 10.1016/j.jsb.2011.12.017

[82] Zivanov, J. et al. New tools for automated high-resolution cryo-EM structure determination in RELION-3. *eLife* **7**, e42166 (2018). 30412051 10.7554/eLife.42166 PMC6250425

[83] Pettersen, E. F. et al. UCSF ChimeraX: structure visualization for researchers, educators, and developers. *Protein Sci.* **30**, 70–82 (2021). 32881101 10.1002/pro.3943 PMC7737788

[84] Kandiah, E. et al. CM01: a facility for cryo-electron microscopy at the European Synchrotron. *Acta Cryst. D Struct. Biol.* **75**, 528–535 (2019). 31205015 10.1107/S2059798319006880 PMC6580229

[85] Zivanov, J., Nakane, T. & Scheres, S. H. W. A Bayesian approach to beam-induced motion correction in cryo-EM single-particle analysis. *IUCrJ* **6**, 5–17 (2019). 30713699 10.1107/S205225251801463X PMC6327179

[86] Rohou, A. & Grigorieff, N. CTFFIND4: Fast and accurate defocus estimation from electron micrographs. *J. Struct. Biol.* **192**, 216–221 (2015). 26278980 10.1016/j.jsb.2015.08.008 PMC6760662

[87] He, S. & Scheres, S. H. W. Helical reconstruction in RELION. *J. Struct. Biol.* **198**, 163–176 (2017). 28193500 10.1016/j.jsb.2017.02.003 PMC5479445

[88] Scheres, S. H. W. Amyloid structure determination in RELION-3.1. *Acta Crystallogr. D Struct. Biol.* **76**, 94–101 (2020). 32038040 10.1107/S2059798319016577 PMC7008511

[89] Chen, S. et al. High-resolution noise substitution to measure overfitting and validate resolution in 3D structure determination by single particle electron cryomicroscopy. *Ultramicroscopy* **135**, 24–35 (2013). 23872039 10.1016/j.ultramic.2013.06.004 PMC3834153

[90] Casañal, A., Lohkamp, B. & Emsley, P. Current developments in Coot for macromolecular model building of electron cryo‐microscopy and crystallographic data. *Protein Sci.* **29**, 1055–1064 (2020). 10.1002/pro.3791 PMC7096722 31730249

[91] Croll, T. I. ISOLDE: a physically realistic environment for model building into low-resolution electron-density maps. *Acta Crystallogr. D Struct. Biol.* **74**, 519–530 (2018). 29872003 10.1107/S2059798318002425 PMC6096486

[92] Brown, A. et al. Tools for macromolecular model building and refinement into electron cryo-microscopy reconstructions. *Acta Crystallogr. D Biol. Crystallogr.* **71**, 136–153 (2015). 25615868 10.1107/S1399004714021683 PMC4304694

[93] Yamashita, K., Palmer, C. M., Burnley, T. & Murshudov, G. N. Cryo-EM single-particle structure refinement and map calculation using Servalcat. *Acta Crystallogr. D Struct. Biol.* **77**, 1282–1291 (2021). 34605431 10.1107/S2059798321009475 PMC8489229

[94] Williams, C. J. et al. MolProbity: more and better reference data for improved all-atom structure validation. *Protein Sci.* **27**, 293–315 (2018). 29067766 10.1002/pro.3330 PMC5734394

[95] Perez-Riverol, Y. et al. The PRIDE database resources in 2022: a hub for mass spectrometry-based proteomics evidences. *Nucleic Acids Res.* **50**, D543–D552 (2022). 34723319 10.1093/nar/gkab1038 PMC8728295

