# Supplementary material for: Tau filaments are tethered within brain extracellular vesicles in Alzheimer’s disease
Source: Nat Neurosci. 2024 Nov 21;28(1):40–8. doi: 10.1038/s41593-024-01801-5 (PMC11706778; doi:10.1038/s41593-024-01801-5)
Supplement: Supplementary file 1 — Supplementary Figs. 1–4. [file 41593_2024_1801_MOESM1_ESM.pdf]

nature neuroscience

Supplementary information https://doi.org/10.1038/s41593-024-01801-5

Tau filaments are tethered within brain extracellular vesicles in Alzheimer's disease

In the format provided by the authors and unedited

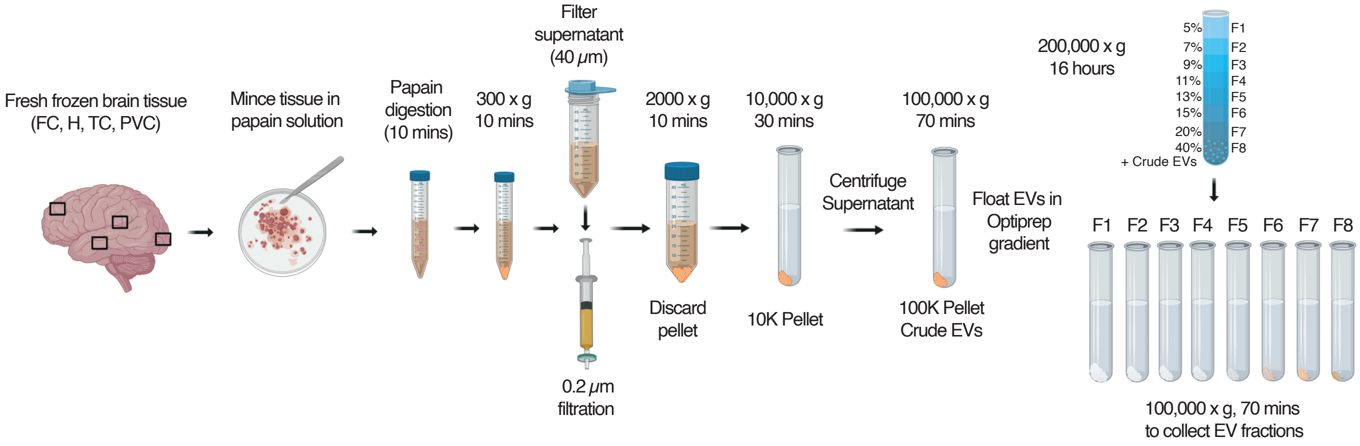

Supplementary Figure 1: Workflow of EV isolation from fresh-frozen post-mortem human brain tissue. Tissue was gently digested in papain, and intact cells and debris were removed by low-speed centrifugation. Crude EVs were generated by high-speed centrifugation and purified by equilibrium density centrifugation in an 8-step Optiprep gradient. Created with BioRender.com.

a

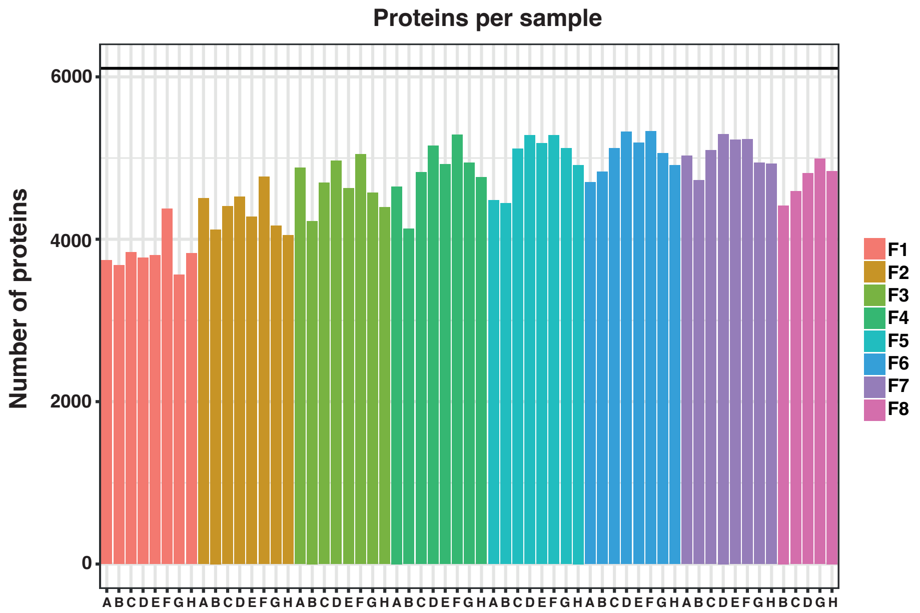

b

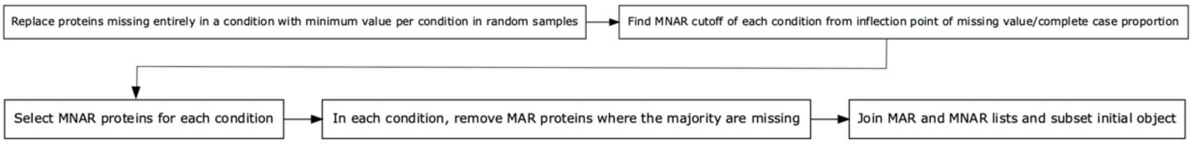

c

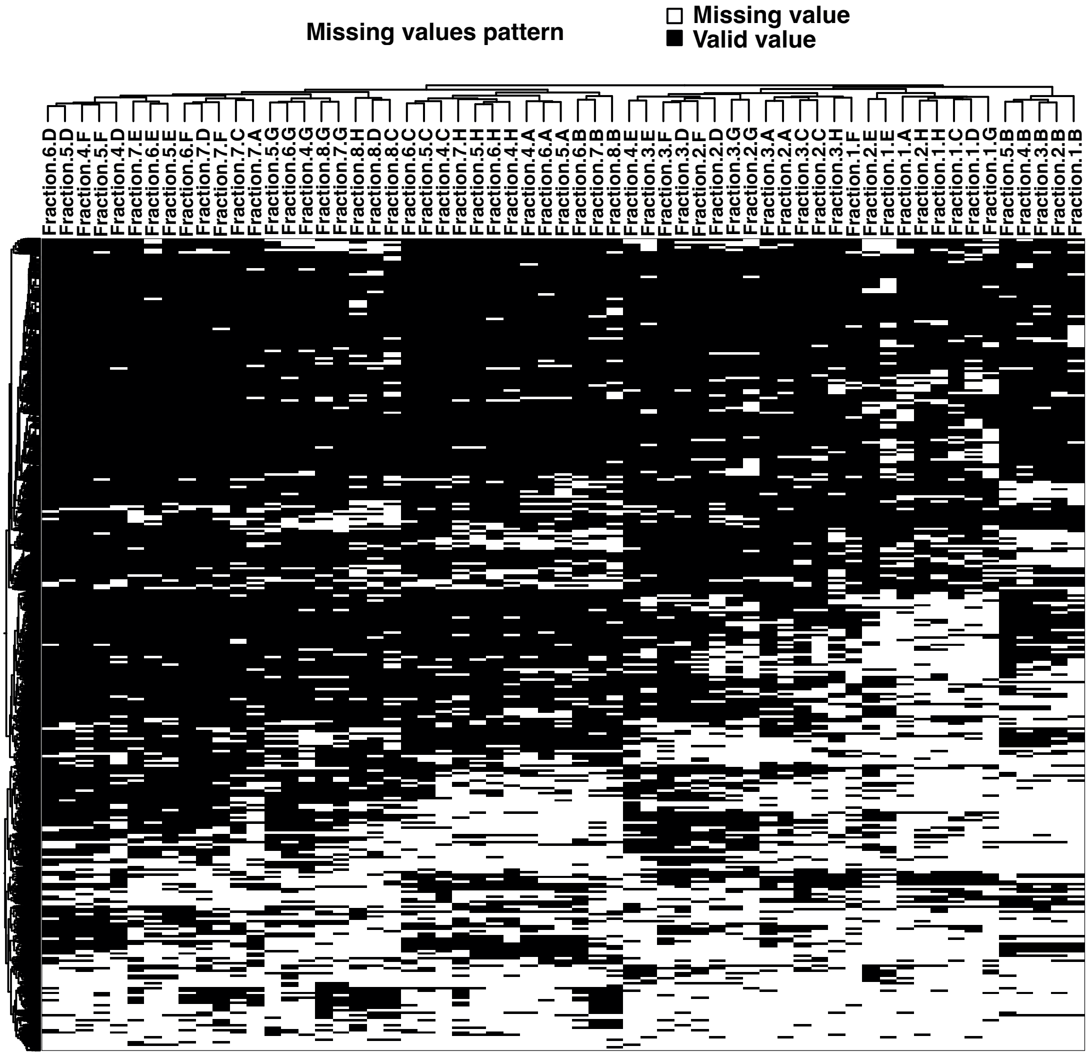

Supplementary Figure 2: Number of proteins and missing values identified by label-free mass spectrometry in each EV fraction from each AD donor. a, Total number of proteins identified in each EV fraction 1–8 from each AD donor A–H. Black horizontal line indicates the total unique number of proteins identified across all samples (6104). **b**, Flowchart describing the data filtering strategy used to produce the final 6054 proteins retained for the imputation pipeline. **c**, Missing values in the raw LFQ dataset for each EV fraction 1–8 from each AD donor A–H. Black rectangles indicate valid values, white rectangles indicate missing values.

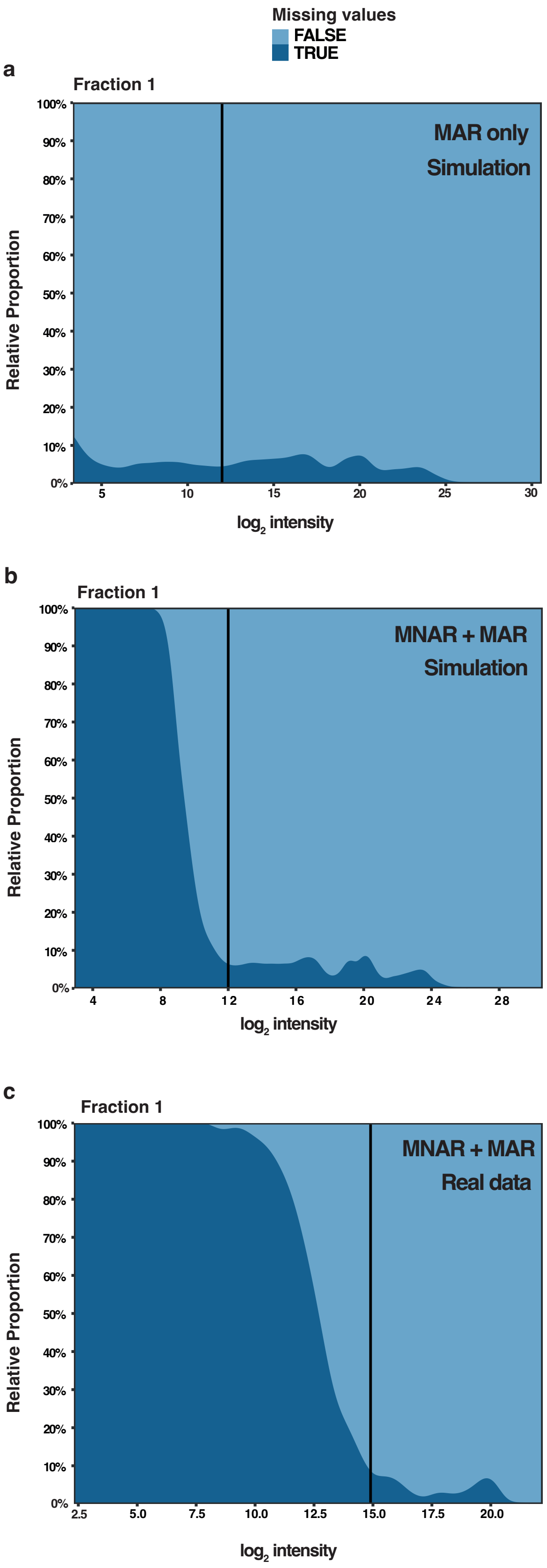

Supplementary Figure 3: Relationship between the relative proportion of true missing values to mean log2 protein intensity values in simulated and real datasets. a, Simulated dataset imputed using a MAR strategy only (MAR values were introduced at 5% of the population). **b**, Simulated dataset imputed using the hybrid MNAR/MAR strategy. The vertical black line indicates the calculated MNAR cutoff value: proteins with log2 intensities less than 12 were imputed with an MNAR strategy, whilst proteins with log2 intensities greater than 12 were imputed with a MAR strategy. The value of 12 maximised benchmarking metrics for the number of true DEPs captured, and the accuracy of their detection (represents the bottom of the steep decline in true missing values). **c**, Real dataset imputed with the hybrid imputation strategy showing that the MNAR cutoff should be set ~15. This point varies only slightly across each fraction and was selected manually to represent the bottom of the steep decline in true missing values.

a

	Imputed	Unimputed	Complete cases
Percent true DEPs captured	92.97	69.07	24.03
Accuracy	93.56	85.80	93.27
Average Adj F p-value of true DEPs	0.0333	0.2976	0.759

b

2925 DEPs from a total of 3000 truly DEP proteins programmed into the simulated dataset

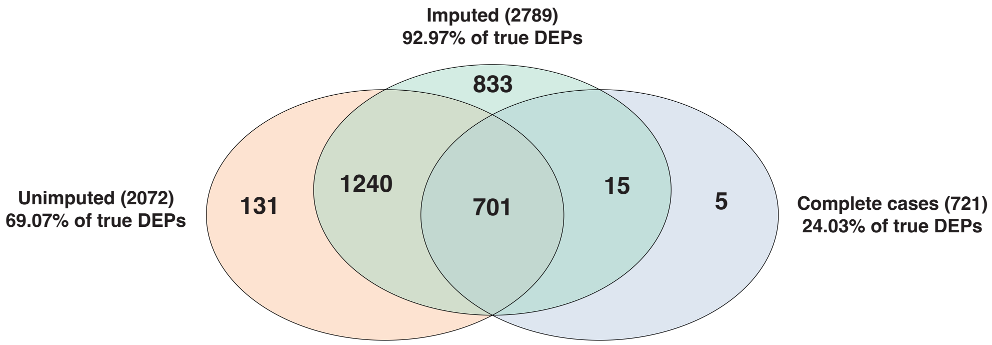

c

3355 DEPs from total 6105 proteins programmed into the simulated dataset

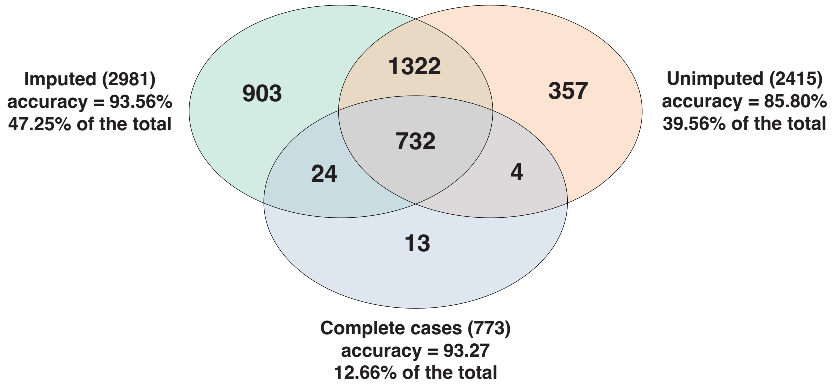

d

DEPs from total 6105 proteins identified in the real dataset

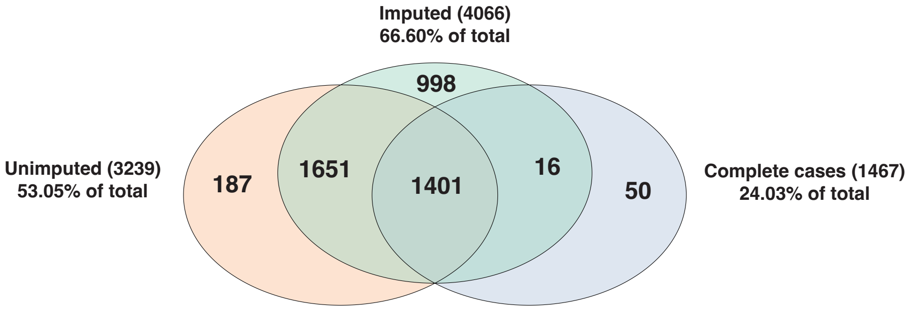

Supplementary Figure 4: Benchmarking of the hybrid imputation strategy. a, For the simulated dataset with 3000 manually programmed DEPs, the percent true DEPs captured, the accuracy, and the average adjusted F p-values in the true DEPs were calculated in the imputed, unimputed, and complete case datasets. **b**, Venn diagram of DEPs captured from 3000 true DEP proteins in imputed, unimputed, and complete cases. **c**, Venn diagram of DEPs captured from 6105 total proteins in imputed, unimputed, and complete cases. These DEPs were compared to those detected in the 3000 true DEPs (panel b) to determine accuracy values to the detection of DEPs. **d**, Venn diagram of DEPs captured from 6105 total proteins in imputed, unimputed, and complete cases from the real dataset. In all cases, DEPs were determined from an ANOVA comparing differences between all possible grouped fraction comparisons.
