# Supplementary material for: Tau filaments are tethered within brain extracellular vesicles in Alzheimer’s disease
Source: Nat Neurosci. 2024 Nov 21;28(1):40–8. doi: 10.1038/s41593-024-01801-5 (PMC11706778; doi:10.1038/s41593-024-01801-5)
Supplement: Supplementary file 2 — Reporting Summary [file 41593_2024_1801_MOESM2_ESM.pdf]

nature portfolio

Corresponding author(s): Karen E. Duff and Benjamin Ryskeldi-Falcon

Last updated by author(s): Sep 2, 2024

Reporting Summary

Nature Portfolio wishes to improve the reproducibility of the work that we publish. This form provides structure for consistency and transparency in reporting. For further information on Nature Portfolio policies, see our Editorial Policies and the Editorial Policy Checklist.

Statistics

For all statistical analyses, confirm that the following items are present in the figure legend, table legend, main text, or Methods section.

n/a	Confirmed	
☐	☒	The exact sample size (*n*) for each experimental group/condition, given as a discrete number and unit of measurement
☐	☒	A statement on whether measurements were taken from distinct samples or whether the same sample was measured repeatedly
☐	☒	The statistical test(s) used AND whether they are one- or two-sided<br>*Only common tests should be described solely by name; describe more complex techniques in the Methods section.*
☐	☒	A description of all covariates tested
☐	☒	A description of any assumptions or corrections, such as tests of normality and adjustment for multiple comparisons
☐	☒	A full description of the statistical parameters including central tendency (e.g. means) or other basic estimates (e.g. regression coefficient) AND variation (e.g. standard deviation) or associated estimates of uncertainty (e.g. confidence intervals)
☐	☒	For null hypothesis testing, the test statistic (e.g. *F*, *t*, *r*) with confidence intervals, effect sizes, degrees of freedom and *P* value noted<br>*Give P values as exact values whenever suitable.*
☒	☐	For Bayesian analysis, information on the choice of priors and Markov chain Monte Carlo settings
☒	☐	For hierarchical and complex designs, identification of the appropriate level for tests and full reporting of outcomes
☒	☐	Estimates of effect sizes (e.g. Cohen's *d*, Pearson's *r*), indicating how they were calculated

Our web collection on statistics for biologists contains articles on many of the points above.

Software and code

Policy information about availability of computer code

Data collection	SerialEM 4.1, EPU 2.14, DIA-NN 1.8
Data analysis	RELION 3.1 and 4.0, CTFFIND 4.1, COOT 0.9.8.2, ISOLDE 1.5, REFMAC 5.8.0387, Servalcat 0.3.0, ChimeraX 1.5, MolProbity 4.5.2, Warp 1.0.9., IMOD 4.11, GraphPad Prism 9, R 4.2.1, DEP 1.20.0, limma 3.54.2, GSVA 1.46.0, Seurat 4.2.0, mice 3.14.0, rmarkdown 2.17, Docker 24.0.7-ce conda 23.3.1, Starparser 1.38, Dynamo2m 0.2.2, Dynamotable 0.2.4, Napari 0.4.12, Napari-tomoslice 0.0.7. Custom code developed in the study can be found here, https://github.com/duff-lab-team/AD-EV-characterisation.

For manuscripts utilizing custom algorithms or software that are central to the research but not yet described in published literature, software must be made available to editors and reviewers. We strongly encourage code deposition in a community repository (e.g. GitHub). See the Nature Portfolio guidelines for submitting code & software for further information.

Data

Policy information about availability of data

All manuscripts must include a data availability statement. This statement should provide the following information, where applicable:

- Accession codes, unique identifiers, or web links for publicly available datasets
- A description of any restrictions on data availability
- For clinical datasets or third party data, please ensure that the statement adheres to our policy

The mass spectrometry proteomics data have been deposited to the ProteomeXchange Consortium via the PRIDE partner repository with the dataset identifier

PXD037708. Data analysis for mass spectrometry proteomics and NTA datasets, and supplemental figures describing the imputation strategy are accessible at https://github.com/duff-lab-team/AD-EV-characterisation. The Homo sapiens proteome is available from the Universal Protein resource (UniProt) under accession code UP000005640. The tomogram shown in Fig. 3a has been deposited to the Electron Microscopy Data Bank (EMDB) under the accession number 16064. Single-particle cryo-EM datasets have been deposited to the Electron Microscopy Public Image Archive (EMPIAR) under the following accession numbers: 11300 for the EV dataset and 11301 for the cellular dataset. Single-particle cryo-EM maps have been deposited to the EMDB under the following accession numbers: 16035 for the EV map and 16039 the cellular map. The atomic models have been deposited to the Protein Data Bank (PDB) under the following accession numbers: 8BGS for the EV model and 8BGV for the cellular model. Any other data are available from the corresponding authors upon request.

Research involving human participants, their data, or biological material

Policy information about studies with human participants or human data. See also policy information about sex, gender (identity/presentation), and sexual orientation and race, ethnicity and racism.

Reporting on sex and gender	7 males and 4 females.
Reporting on race, ethnicity, or other socially relevant groupings	*Please specify the socially constructed or socially relevant categorization variable(s) used in your manuscript and explain why they were used. Please note that such variables should not be used as proxies for other socially constructed/relevant variables (for example, race or ethnicity should not be used as a proxy for socioeconomic status).* *Provide clear definitions of the relevant terms used, how they were provided (by the participants/respondents, the researchers, or third parties), and the method(s) used to classify people into the different categories (e.g. self-report, census or administrative data, social media data, etc.)* *Please provide details about how you controlled for confounding variables in your analyses.*
Population characteristics	See Extended Data Table 1. Between 47 and 89+ years-of-age. No neurodegenerative disease associated mutations. Clinical and neuropathological diagnoses of Alzheimer's disease.
Recruitment	Selected based on availability and neuropathological examination.
Ethics oversight	All human AD specimens were sourced from the New York Brain Bank (NYBB) at Columbia University (Alzheimer's Disease Research Center) and the University of Miami Brain Endowment Bank. Their use in this study was approved by the ethical review processes at each institution.

Note that full information on the approval of the study protocol must also be provided in the manuscript.

Field-specific reporting

Please select the one below that is the best fit for your research. If you are not sure, read the appropriate sections before making your selection.

☒ Life sciences ☐ Behavioural & social sciences ☐ Ecological, evolutionary & environmental sciences

For a reference copy of the document with all sections, see nature.com/documents/nr-reporting-summary-flat.pdf

Life sciences study design

All studies must disclose on these points even when the disclosure is negative.

Sample size	For cryo-ET, temporal cortex and hippocampus from an individual with Alzheimer's disease. For cryo-EM, temporal cortex, hippocampus and amygdala from two individuals with Alzheimer's disease. For mass spectrometry, head of hippocampus, hippocampus, temporal cortex, and frontal cortex were used from 5 male and 3 female individuals with Alzheimer's Disease (aged 67-89). Samples were chosen based on availability and neuropathological examination.
Data exclusions	Pre-established common image classification procedures (Scheres 2012. J. Struc. Biol. 180, 519-530) were employed to select particle images with the highest resolution content in the cryo-EM reconstruction process. Details of the number of selected images are given in Extended Data Table 2. For the mass spectrometry analysis, EV fractions 8A, F, and E were excluded from the analysis as significantly less material was isolated from those donors.
Replication	All attempts at replication were successful. At least three independent biological repeats per experiment where representative data are shown, as described in the main text.
Randomization	Randomisation was not performed. As the samples were limited by brain availability, randomisation would not have reduced any bias in this study.
Blinding	The investigators were not blinded to allocation during experiments and outcome assessment. The perceived risk of detection/performance bias was deemed negligible.

Reporting for specific materials, systems and methods

We require information from authors about some types of materials, experimental systems and methods used in many studies. Here, indicate whether each material, system or method listed is relevant to your study. If you are not sure if a list item applies to your research, read the appropriate section before selecting a response.

Materials & experimental systems

n/a	Involved in the study
☐	☒ Antibodies
☐	☒ Eukaryotic cell lines
☒	☐ Palaeontology and archaeology
☐	☒ Animals and other organisms
☒	☐ Clinical data
☒	☐ Dual use research of concern
☒	☐ Plants

Methods

n/a	Involved in the study
☒	☐ ChIP-seq
☒	☐ Flow cytometry
☒	☐ MRI-based neuroimaging

Antibodies

Antibodies used

Rabbit monoclonal anti-Annexin A2 clone D11G2 Cell Signaling Technology Cat# 8235; RRID: AB_11129437; used at 1/1000
Mouse monoclonal anti-Flotillin 1 BD Transduction Cat# 610821; RRID: AB_398140; used at 1/1000
Rabbit monoclonal anti-CD81, clone D3N2D Cell Signaling Technology Cat# 56039; RRID: AB_2924772; used at 1/1000
Mouse monoclonal anti-LAMP2, clone H4B4 Santa Cruz Cat# sc-18822; RRID: AB_626858; used at 1/1000
Rabbit polyclonal anti-LAMP1 Abcam Cat# ab62562; RRID:AB_2134489; used at 1/250
Rabbit polyclonal, anti-VDAC Cell Signaling Cat# 48665; RRID: AB_2272627; used at 1/1000
Mouse monoclonal anti-Lamin A/C, clone E-1 Santa Cruz Cat# sc-376248; RRID: AB_10991536; used at 1/1000
Mouse monoclonal anti-tau (Tau13) BioVision Cat# 3453-100; RRID: AB_592814; used at 1/1000
Mouse monoclonal anti-tau (HT7) Invitrogen Cat# MN1000; RRID: AB_2314654; used at 1/1000
Rabbit polyclonal anti-tau (TauC) DAKO Cat# A0024; RRID: AB_10013724; used at 1/5000
Mouse monoclonal anti-PHF tau (PHF1) Gift from Peter Davies Cat# PHF1; RRID: AB_2315150; used at 1/250
Mouse monoclonal anti-EEA1, clone 14 BD Biosciences Cat# 610457; RRID: AB_397830; used at 1/250
Mouse monoclonal anti-β-Actin-FITC, clone AC-15 Sigma-Aldrich Cat#F3022; RRID: AB_476970; used at 1/1000
Mouse monoclonal anti-tau MC1 Gift from Peter Davies Cat# MC1, RRID:AB_2314773; used at 1/250
Rabbit polyclonal anti-phospho tau-Thr217 Thermo Fisher Scientific Cat# PA5-37639, RRID:AB_2554247 Verified by cell treatment (retinoic acid and okadaic acid); used at 1/250
Rabbit polyclonal anti-phospho tau-Ser422 Thermo Fisher Scientific Cat# 44-764G, RRID:AB_2533748 Verified by cell treatment (phosphopeptide blocking and lambda phosphatase treatment); used at 1/250

Validation

Validation described on supplier websites, PHF1 antibody described in: PMID: 1370450, MC1 antibody described in: PMID: 9349554.

Eukaryotic cell lines

Policy information about cell lines and Sex and Gender in Research

Cell line source(s)

Tau RD P301S FRET Biosensor cells (ATCC CRL-3275), HEK293T cells (ATCC crl-3216)

Authentication

ATCC CRL-3275 is authenticated by ATCC. Parental ATCC crl-3216 cell line is authenticated by ATCC; modified crl-3216 cell lines expressing P301S 1N4R tau fused to YFP (seeded or unseeded with human tau PHFs) have not been authenticated.

Mycoplasma contamination

All cell lines tested negative for mycoplasma contamination.

Commonly misidentified lines
(See ICLAC register)

No commonly misidentified cell lines were used in the study.

Animals and other research organisms

Policy information about studies involving animals; ARRIVE guidelines recommended for reporting animal research, and Sex and Gender in Research

Laboratory animals

6-month-old PS19 JAX mice #008169; https://www.jax.org/strain/008169

Wild animals

No wild animals were used in the study.

Reporting on sex

Sex was not considered a relevant variable in this study; male mice were used to limit possible effects of cycling hormones.

Field-collected samples

No field collected samples were used in the study.

Ethics oversight

All experiments were performed in accordance with protocols approved by the Institutional Animal Care and Use Committee at Columbia University (NYC).

Note that full information on the approval of the study protocol must also be provided in the manuscript.

Plants

Seed stocks

Report on the source of all seed stocks or other plant material used. If applicable, state the seed stock centre and catalogue number. If plant specimens were collected from the field, describe the collection location, date and sampling procedures.

Novel plant genotypes

Describe the methods by which all novel plant genotypes were produced. This includes those generated by transgenic approaches, gene editing, chemical/radiation-based mutagenesis and hybridization. For transgenic lines, describe the transformation method, the number of independent lines analyzed and the generation upon which experiments were performed. For gene-edited lines, describe the editor used, the endogenous sequence targeted for editing, the targeting guide RNA sequence (if applicable) and how the editor was applied.

Authentication

Describe any authentication procedures for each seed stock used or novel genotype generated. Describe any experiments used to assess the effect of a mutation and, where applicable, how potential secondary effects (e.g. second site T-DNA insertions, mosiacism, off-target gene editing) were examined.
