# Supplementary figures and images for: Tau filaments are tethered within brain extracellular vesicles in Alzheimer’s disease
Source: Nat Neurosci. 2024 Nov 21;28(1):40–8. doi: 10.1038/s41593-024-01801-5 (PMC11706778; doi:10.1038/s41593-024-01801-5)

Fig 1a:

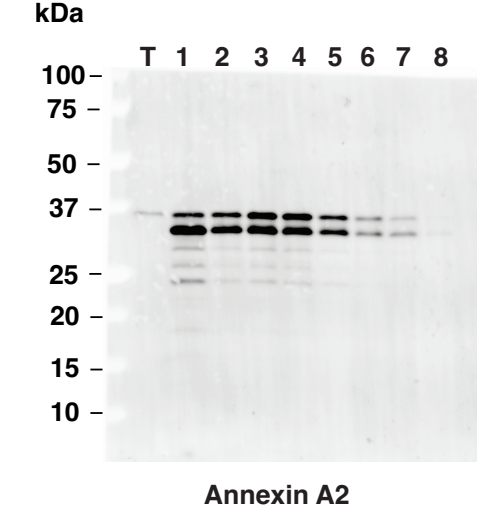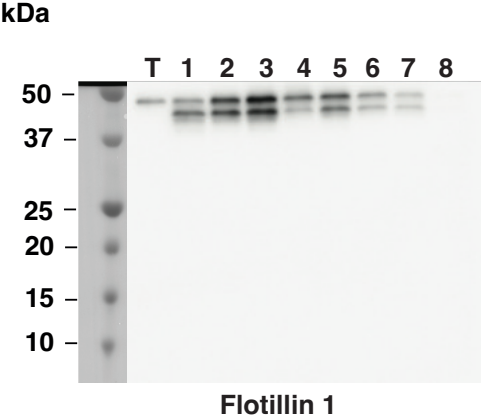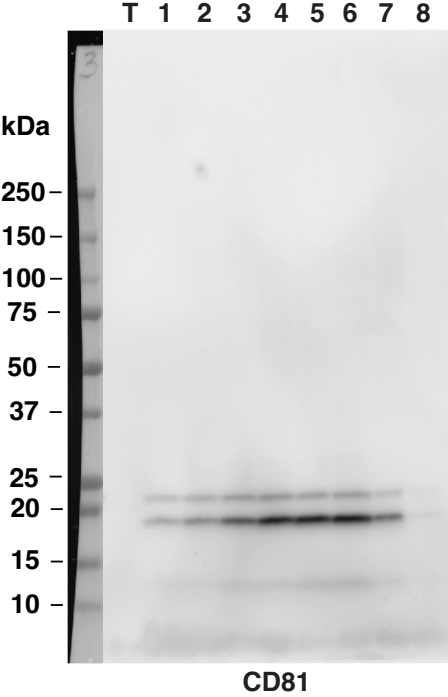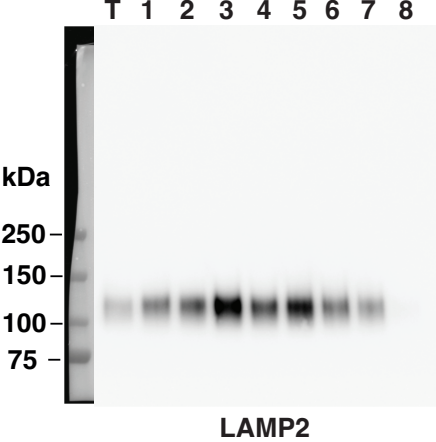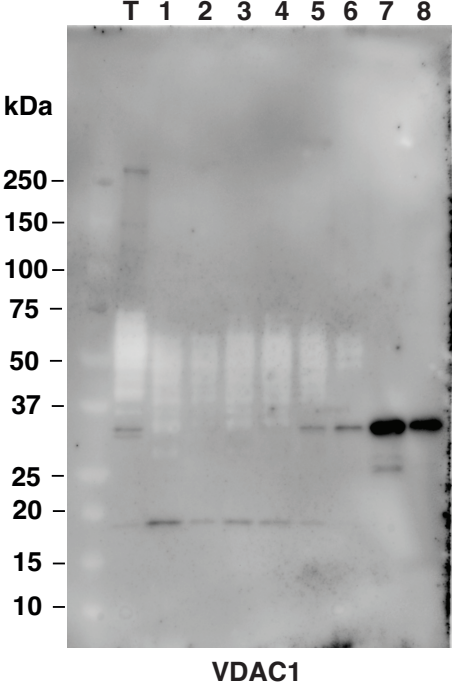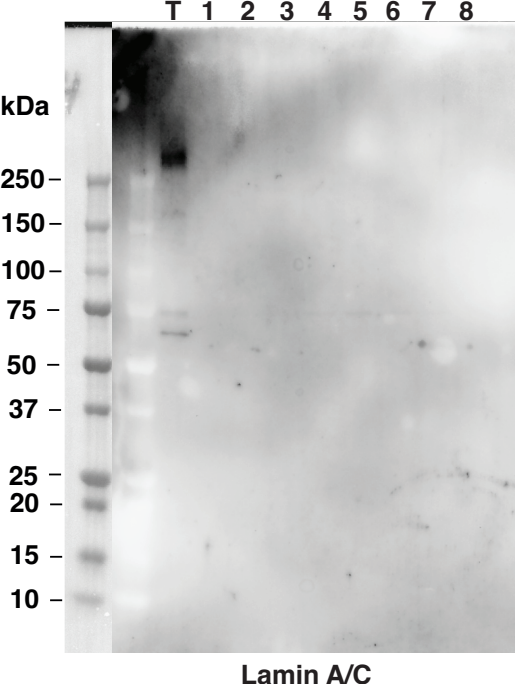

Fig 2:

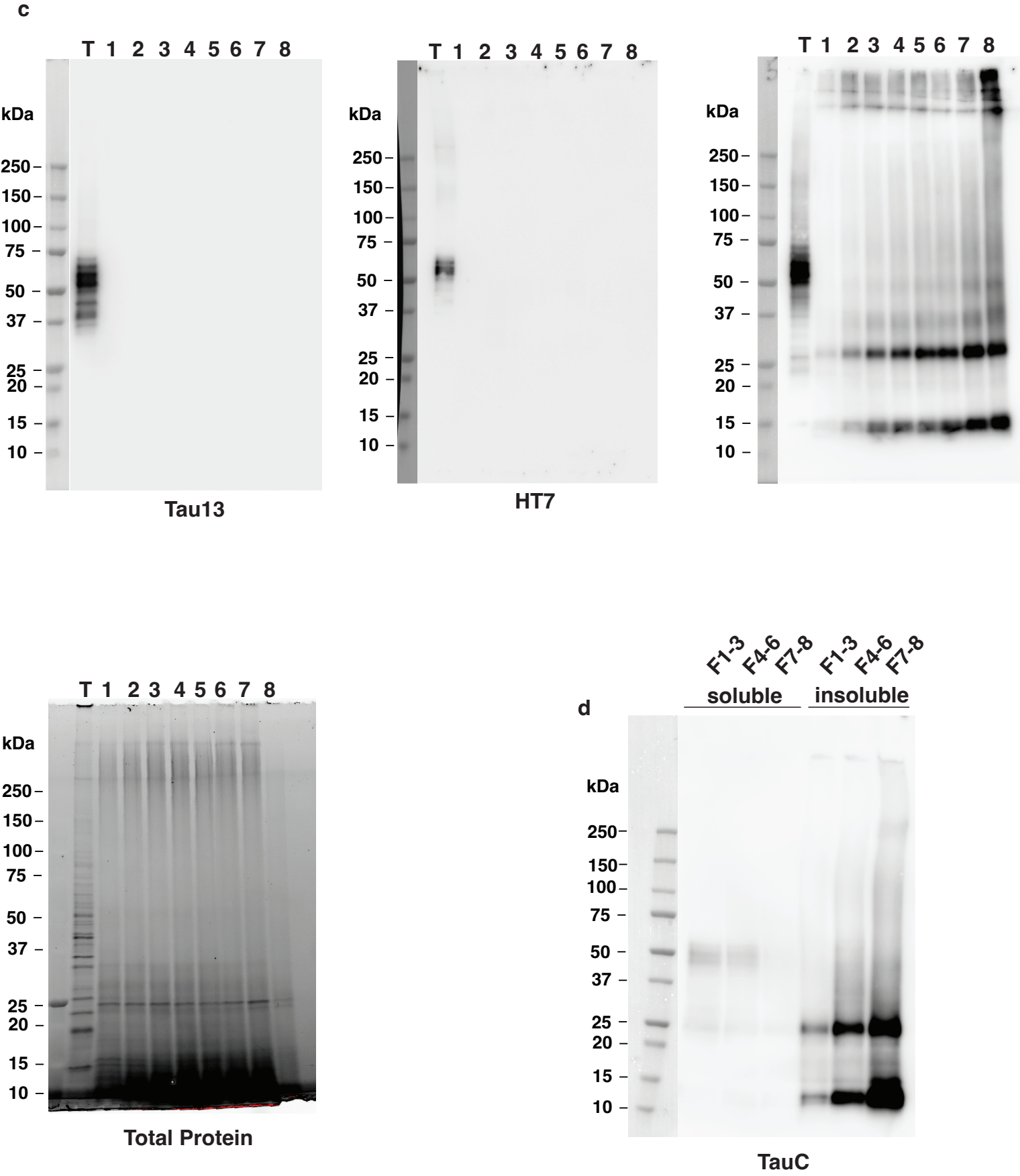

Fig 3c:

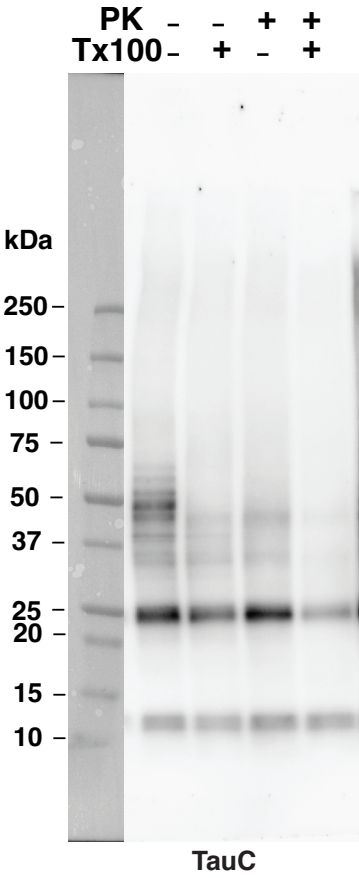

Fig 4c:

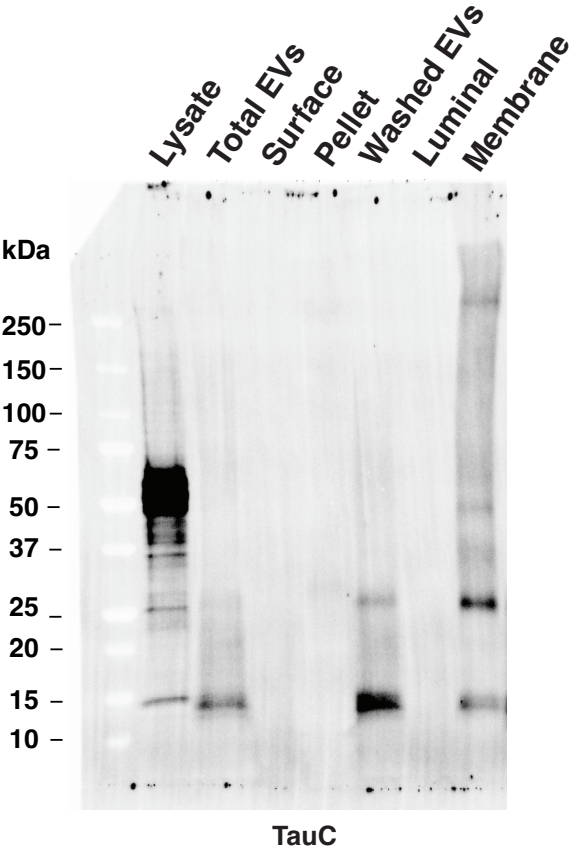

Ext Data Fig 4c:

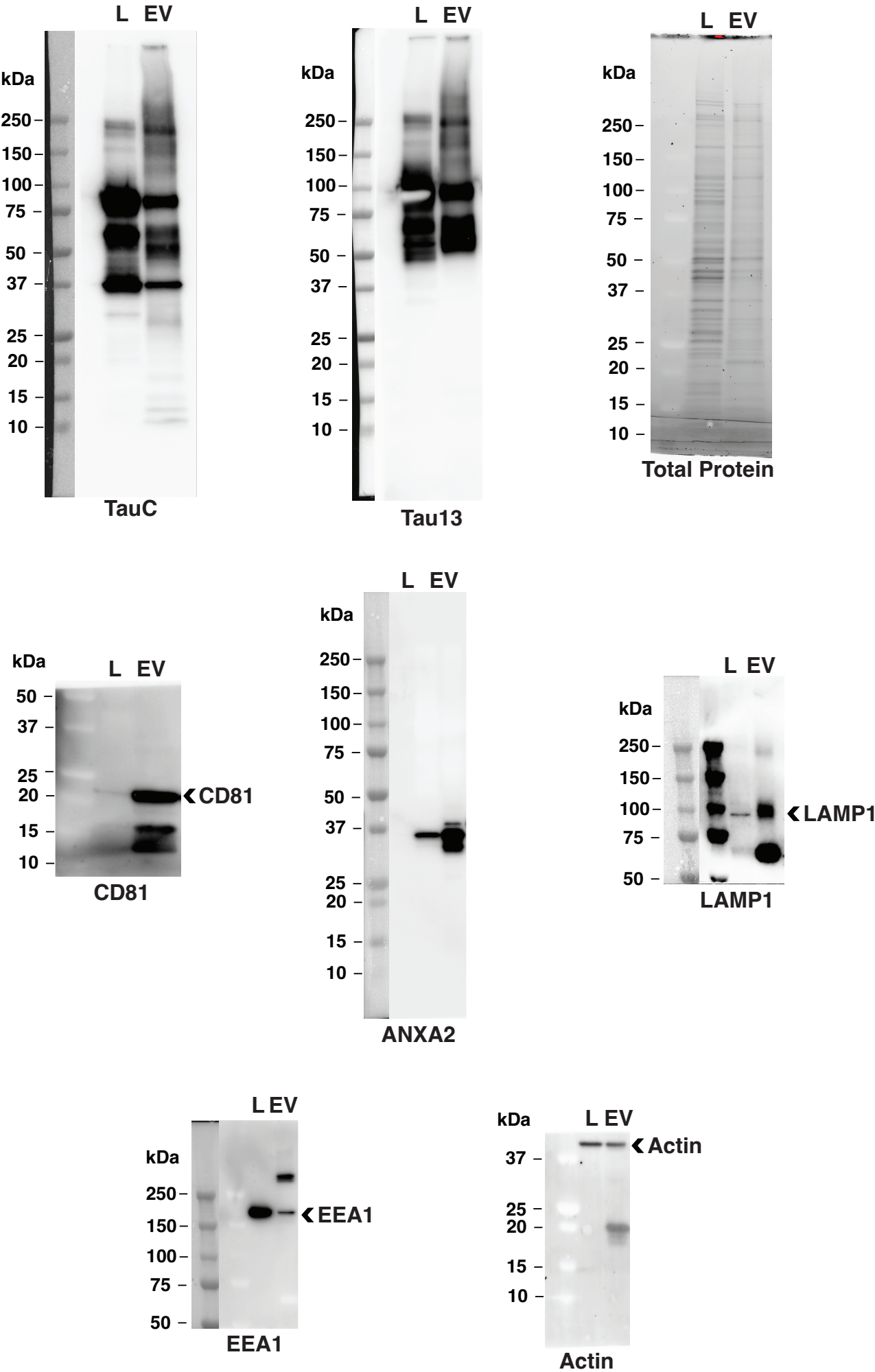

Ext Data Fig 5:

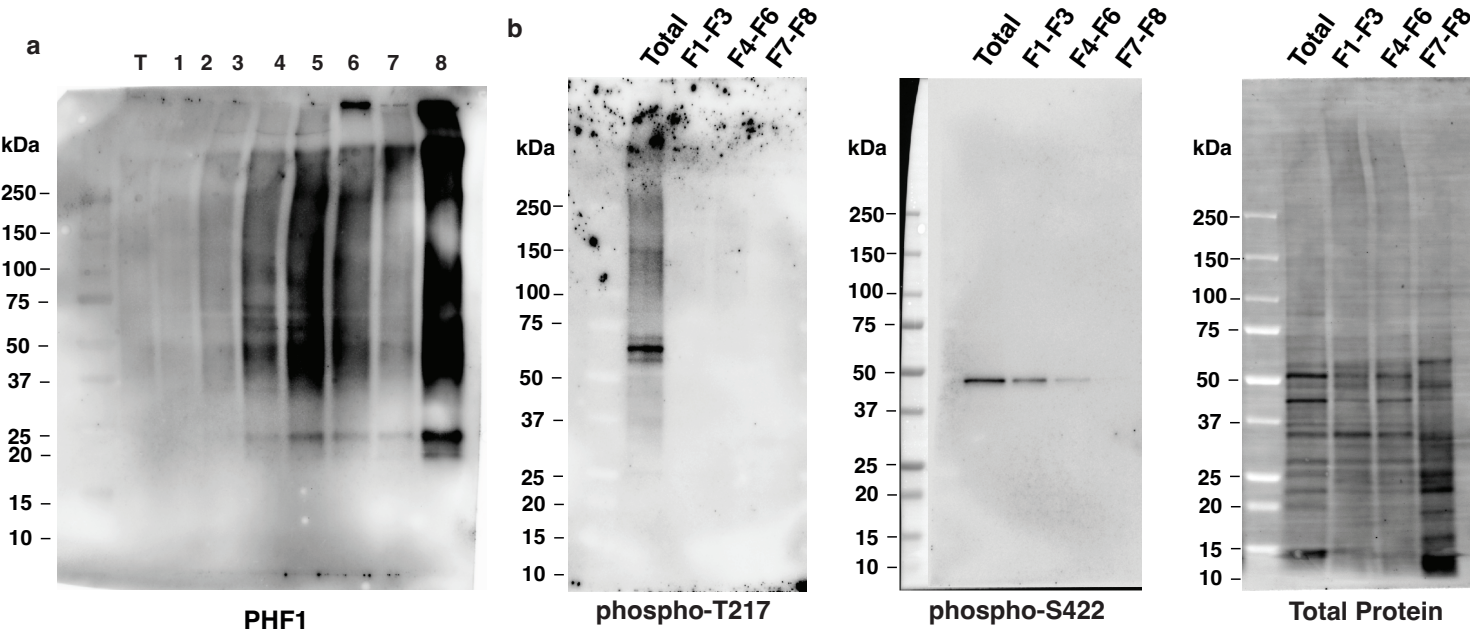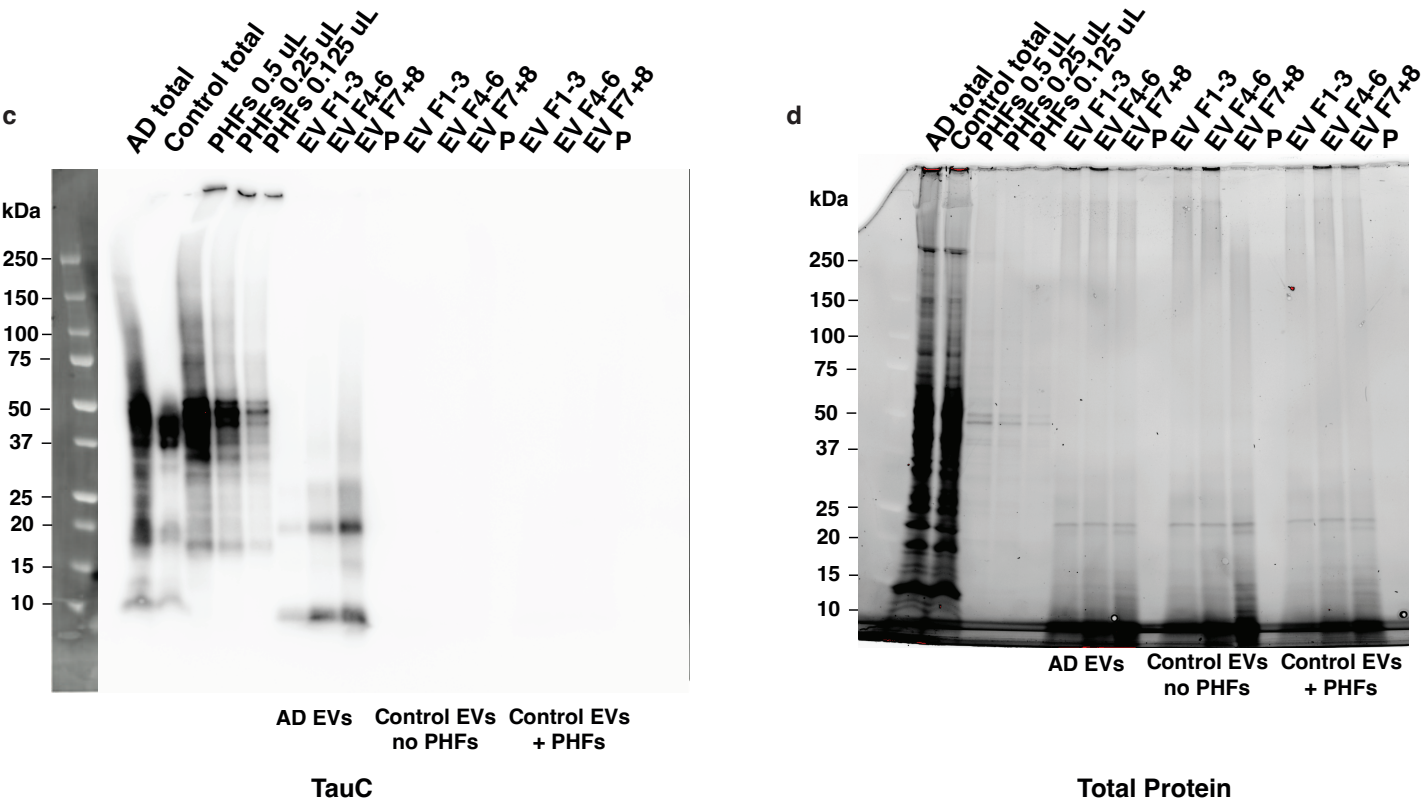

Supplement: Supplementary file 4 — Unprocessed western blots for Figs. 1, 2c,d, 3c and 4c and Extended Data Figs. 4c and 5. [file 41593_2024_1801_MOESM4_ESM.pdf]
